# Avian *Paramyxovirus* Type 1 in Egypt: Epidemiology, Evolutionary Perspective, and Vaccine Approach

**DOI:** 10.3389/fvets.2021.647462

**Published:** 2021-07-15

**Authors:** Shimaa M. G. Mansour, Reham M. ElBakrey, Fakry F. Mohamed, Esraa E. Hamouda, Mona S. Abdallah, Ahmed R. Elbestawy, Mahmoud M. Ismail, Hanan M. F. Abdien, Amal A. M. Eid

**Affiliations:** ^1^Department of Virology, Faculty of Veterinary Medicine, Zagazig University, Zagazig, Egypt; ^2^Department of Avian and Rabbit Medicine, Faculty of Veterinary Medicine, Zagazig University, Zagazig, Egypt; ^3^Department of Avian and Rabbit Medicine, Faculty of Veterinary Medicine, Suez Canal University, Ismailia, Egypt; ^4^Department of Poultry and Fish Diseases, Faculty of Veterinary Medicine, Damanhur University, Damanhur, Egypt; ^5^Department of Poultry Diseases, Faculty of Veterinary Medicine, Kafrelsheikh University, Kafr El-Sheikh, Egypt

**Keywords:** APMV-1, Newcastle disease virus, phylogenetic analysis, deduced amino acid, cleavage site, fusion protein, genotypes

## Abstract

*Avian orthoavulavirus* 1, formerly known as avian paramyxovirus type-1 (APMV-1), infects more than 250 different species of birds. It causes a broad range of clinical diseases and results in devastating economic impact due to high morbidity and mortality in addition to trade restrictions. The ease of spread has allowed the virus to disseminate worldwide with subjective virulence, which depends on the virus strain and host species. The emergence of new virulent genotypes among global epizootics, including those from Egypt, illustrates the time-to-time genomic alterations that lead to simultaneous evolution of distinct APMV-1 genotypes at different geographic locations across the world. In Egypt, the Newcastle disease was firstly reported in 1947 and continued to occur, despite rigorous prophylactic vaccination, and remained a potential threat to commercial and backyard poultry production. Since 2005, many researchers have investigated the nature of APMV-1 in different outbreaks, as they found several APMV-1 genotypes circulating among various species. The unique intermingling of migratory, free-living, and domesticated birds besides the availability of frequently mobile wild birds in Egypt may facilitate the evolution power of APMV-1 in Egypt. Pigeons and waterfowls are of interest due to their inclusion in Egyptian poultry industry and their ability to spread the infection to other birds either by presence of different genotypes (as in pigeons) or by harboring a clinically silent disease (as in waterfowl). This review details (i) the genetic and pathobiologic features of APMV-1 infections in Egypt, (ii) the epidemiologic and evolutionary events in different avian species, and (iii) the vaccine applications and challenges in Egypt.

## Introduction

Newcastle disease (ND) is a highly contagious notifiable viral disease with significant clinical impact and heavy economic losses to the poultry industry worldwide (1). It is on the list A of OIE and ranked as the second-highest endemic disease in many countries (2). In developing or developed countries where chickens are raised in small household or commercial sectors, ND has a significant economic impact on poultry sector due to high mortality rates (up to 100%), decreased productivity, and disease prevention and control expenses as well as trade restrictions (1).

Faulty vaccination programs and incorrect administration, transport, and storage of vaccine, as well as concurrent infection, may play a role in maximizing the losses and impact of ND field challenge. Heterogeneous genotype cross-protection is controversial. Repeated ND virus (NDV) infections even in vaccinated birds could be attributed to improper vaccination and immune suppression along with viral mutation (3). The molecular epidemiology and sequence analysis of NDV in Egypt are important to determine the current situation and available control measures (4). In Egypt, NDV was firstly identified in 1947 (5) by virus isolation in embryonated chicken eggs (ECEs) and then identification using hemagglutination inhibition (HI) assay. Despite extensive vaccination programs against ND in both commercial and backyard poultry flocks across Egypt, many outbreaks have occurred since then, resulting in huge economic losses. This raises the inquiries about the genetic diversity of the indigenous strains as well as the feasibility of commercial vaccines on protection against circulating NDVs (6). Besides, mixed infections in birds with NDV, and other viral infections such as infectious bronchitis virus (IBV), avian influenza viruses (AIVs) (H5Nx or H9N2), or avian reovirus, usually aggravate losses (4, 7–10).

The urgent and long-awaited questions had affirmative answers: Do different genotypes change the keys to deal with prevention of ND and change the strategies of making effective vaccines in protecting against NDV? Are traditional vaccines on their way to extinction? For better answers, there is a need to analyze the outcome of available studies to achieve the most of the divine gift that concerns natural lentogenic and avirulent strains for protection against circulating velogenic NDVs. This review provides a historical overview of NDV status in Egypt, concerning the pathobiology and epidemiology of NDV and timely genetic and evolutionary insights in the virus genome, along with the background and rationale of vaccine strategies and challenges, aiming to develop insights toward solving the NDV endemic situation in Egypt.

## Etiology

The avian *orthoavulavirus* 1, also known as NDV, is an enveloped, negative-sense, single-stranded RNA virus that belongs to the family *Paramyxoviridae* under the order *Mononegavirales* (11). The viral genome is around 15,200 bp in length and encodes for six different protein, namely, nucleocapsid protein (NP), phosphoprotein (P), matrix (M) protein, large RNA polymerase (L), fusion (F) protein, and hemagglutinin–neuraminidase (HN). The F and HN surface glycoproteins are involved in the antigenicity and pathogenicity of NDV (12). Two other proteins (V and W) could also be coded through P protein mRNA editing (13).

Based on the phylogenetic analysis of F gene sequences, the NDV can be divided into two classes (I and II): class I usually includes avirulent viruses, and their natural reservoir is aquatic wild birds (14), whereas class II consists of at least 20 genotypes (I–XXI, as genotype XV that contains only recombinant sequences was excluded from the final analyses) and includes both avirulent and virulent strains (15–17). According to its pathogenicity, NDV is categorized into five pathotypes: asymptomatic enteric, lentogenic, mesogenic, neurotropic velogenic, and viscerotropic velogenic (18). One major determinant of NDV virulence is the F protein cleavage site (19). The lentogenic strains have a monobasic amino acid (aa) motif 112GR/K-Q-GR↓L117, while velogenic and mesogenic strains have a multi-basic aa motif, 112R/G/K-R-Q/KK/R-R↓F117 (20).

Pigeon paramyxovirus type-1 (PPMV-1) is an antigenic variant of *Avian orthoavulavirus* 1 and is known to infect pigeons, doves, wild birds, and domestic poultry. It induces nervous manifestations similar to the nervous form of ND, with few evident respiratory signs (21, 22). PPMV-1 can be differentiated from classical NDV by HI test, monoclonal antibodies, and restriction enzyme analysis of F gene. Phylogenetically, PPMV-1 isolates are classified as a distinct sub-genotype within genotype VI of class II (sub-genotype VIb) (23). Four major panzootics of ND have been reported in different avian species (24). The third outbreak, mostly affecting pigeons and doves, was caused by PPMV-1, which originated in the Middle East (Iraq) in the late 1970s and then spread rapidly to Europe (25, 26). Now, PPMV-1 is endemic in domestic and feral pigeons in many areas of the world including the USA and Europe (27). Despite the control measures, PPMV-1 remained enzootic in several countries, including Egypt, causing economic losses (7, 22).

## Pathobiological and Epidemiological Perspectives of Newcastle Disease Virus in Different Avian Species

The ND affects a wide range of domestic and wild birds, with greatly varying pathogenicity, spanning from peracute disease (with up to 100% mortality) to asymptomatic disease. Such variability makes it challenging to sort ND as a single clinicopathological entity. Over 250 species of birds are susceptible to NDV infection. It is known that PPMV-1 attacks mainly pigeons and less frequently chickens. In addition, there are cases of virus infection in birds kept in captivity and wild birds, including pheasants, partridges, falcons, swans, cockatoos, blackbirds, and budgerigars (28–32).

**Figure 1:**
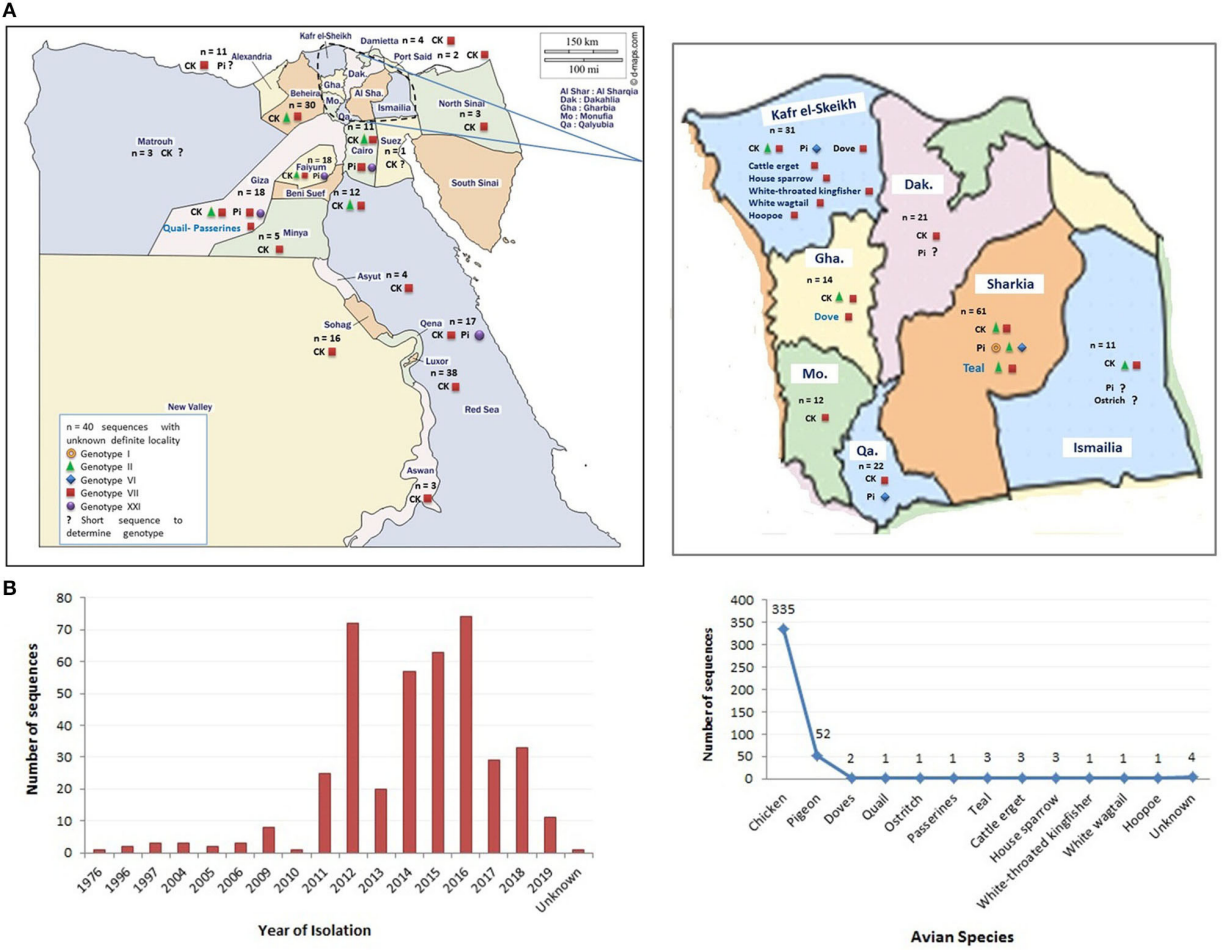
The distribution of Newcastle disease virus (NDV) strains (sequences) in Egypt. **(A)** The geographical detection of NDV in different Egyptian provinces (data obtained from GenBank in early December 2020). **(B)** The grouping of detected NDV strains according to year of detection and species of origin. Color codes are indicated in the figures.

In this review, we represent a nationwide prospective of NDV in Egypt, including the ecology and prevalence in different avian species (Figure 1), vaccine approaches, and molecular aspects of previously isolated NDV strains, which shall hopefully reveal the potential causes of NDV emergence and dissemination in Egypt.

### Chickens

Chickens are the most commonly affected avian species. Clinically, five NDV pathotypes were categorized. Additionally, *in vivo* testing in embryos or chickens/pigeons can be done to determine strain pathogenicity, including mean death time (MDT), intravenous (IV) pathogenicity index (IVPI), and intracerebral pathogenicity index (ICPI). The MDT is the time to death, measured in hours, after inoculation of ECEs. The MDT for the NDV strains, <60, 60–90, or >90 h, was considered velogenic, mesogenic, or lentogenic strains, respectively (2, 33). The IVPI involves scoring illness (0 = normal; 1 = sick; 2 = paralyzed or nervous signs; and 3 = death) after IV inoculation of 6-week-old chickens. The velogenic strains have an IVPI score of 2–3, mesogenic 0–0.5, and lentogenic of zero (34). Now, the definitive *in vivo* assessment of NDV virulence is based on the ICPI test. It is considered as the most sensitive and widely used test for measuring the virulence in ten 1-day-old chicks (2, 35). The ICPI involves scoring sick or dead (0 = normal; 1 = sick; and 2 = dead). Scores with an ICPI of 1.5–2 is considered velogenic, 1–1.5 mesogenic, and 0–0.5 lentogenic. ICPI score of ≥0.7 is considered “notifiable” to the OIE (2). Particular drawbacks of pathogenicity indices are in the interpretation of pathotype results. A previous study (36) reported 10 NDV isolates from pigeons to have ICPI values (1.2–1.45) and IVPI values (0–1.3), classifying the isolates as virulent. However, MDT was low (98 h) in lentogenic strains. Indeed, not all virulent strains have an MDT <60 h. The *in vivo* tests on NDV isolates from species other than chickens can present some problems and may not produce accurate interpretations until passaged in chickens or ECEs (37). The course of the NDV infection can vary widely depending on the virulence of the virus. Several studies were conducted in Egypt on the *in vivo* pathogenicity of NDV field isolates (Table 1). Earlier, in a surveillance among more than 100 chicken flocks at Sharkia Province in late 1980s, 26 NDVs were isolated, velogenic (*n* = 15), mesogenic (*n* = 9), and lentogenic (*n* = 2) isolates, from vaccinated birds, besides one mesogenic from egg shell and other lentogenic from drinking water. The velogenic NDVs showed IVPI (1.5–1.8) in chickens; however, higher figures of ICPI (2.3–3.7) were recorded in pigeon squabs (38). The values of pathogenicity indices of Egyptian NDV isolates (Table 1) revealed that the majority of NDV strains were velogenic. The MDT (36–60 h), IVPI values of 1.5–3, and ICPI scores (1.6–2.0) are indicative for velogenic nature NDV isolates. However, for mesogenic strains, the MDT and ICPI were >60 h and 0.5–1.5, respectively. Besides, 96–108 h and 0.38–0.44 for MDT and ICPI, respectively, indicate the lentogenic nature of strains. The aforesaid findings are consistent with the sequence analysis of F protein cleavage site. In contrast, Nagy et al. (51) characterized 13 NDV isolates from vaccinated chicken flocks during 2014–2017; two of them, Ck/ME3/Eg/16 and Ck/ME5/Eg/16, had the 112GRQGRL117 cleavage motif characteristic to lentogenic strains, although they showed MDT <36–48 h and high ICPI (1.89–2.00) congruent for velogenic pathotype. Similarly, Naguib et al. (53) recorded that the sequence of isolate R1973/11 represents a cleavage site of lentogenic viruses; however, it had an ICPI of 1.88, clearly categorizing the isolate as velogenic. The applied molecular pathotyping by RT-qPCR using the primers and probes specific for avirulent and virulent pathotypes revealed that two Egyptian NDV isolates during 2011 were positive for lentogenic and virulent pathotypes, indicating mixed infection.

**Table 1:** Pathogenicity indices of NDV strains isolated from Egypt.

**Year**	**Origin of virus**	**Pathogenicity index**			**Pathotype**	**References**
		**MDT (h)**	**IVPI**	**ICPI**		
1986–1988	Chicken		1.5–1.8	2.3–3.7^*^	Velogenic	(38)
			0.07–0.81	0.4–1.7^*^	Mesogenic	
			–	0	Lentogenic	
1996–2005	Chicken	36–57	–	–	Velogenic	(39)
		96–104	–	–	Lentogenic	
2005	Chicken	60	2–3	–	Velogenic	(40)
2005	Chicken	55	2.5	1.75	Velogenic	(41)
2006	Chicken	50–60	2.1–2.25	1.6–1.8	Velogenic	(42)
2011–2012	Chicken	–	–	1.4–2	Mesogenic Velogenic	(43)
2011–2012	Chicken	–	–	1.96	Velogenic	(44)
2011–2014	Chicken	≤ 48	–	1.66–1.73	Velogenic	(45)
		108	–	–	Lentogenic	
2012–2014	Chicken	48	–	1.625	Velogenic	(46)
		96	–	0.4375	Lentogenic	
2013–2014	Chicken			>1.5	Velogenic	(46)
2014	Chicken	48	–	1.66	Velogenic	(47)
		96	–	0.44	Lentogenic	
2012–2015	Chicken	–	–	>1.5–2	Velogenic	(48)
		–	–	0.5–1.5	Mesogenic	
2014–2015	Chicken	48	–	1.1–1.89	Velogenic	(49)
		<60	–	0.9	Mesogenic	
		96	–	0.38	Lentogenic	
2012–2016	Chicken	–	–	1.66–1.73	Velogenic	(50)
2014–2017	Chicken	≤ 36–48	–	1.88–2.00	Velogenic	(51)
2015–2018	Chicken	–	–	1.60–1.74	Velogenic	(52)
2015–2019	Chicken	–	–	1.70–1.98	Velogenic	(10)
2011–2014	Chicken	–	–	0.9	Mesogenic	(53)
		–	–	1.88	Velogenic	
2014	Wild pigeons	86	–	1.2	Mesogenic	(54)
2015	Pigeon	–	–	1.31	Mesogenic	(55)
2016	Pigeon	64–69	0	1.41–1.51	Mesogenic	(56)
1976	Migratory birds^a^	59.2–77.6	–	1.95–3.02^*^		(57)
Unknown	Quail	70–80	–	–	Mesogenic	(58)
2016	Quail	64.1		1.79	Velogenic	(59)
2016	Cattle egret	63.2		1.6	Velogenic	
2016	Teal	63.8–65.0		1.72–1.83	Velogenic	

*NDV, Newcastle disease virus; ICPI, intracerebral pathogenicity index; IMPI, intramuscular pathogenic index; IVPI, intravenous pathogenicity index; MDT, mean death time*.

* *ICPI in pigeon squabs*.

a *The author determined ICPI (1.95–3.02) and IMPI (0.14–1.95) in pigeons and IMPI (1.8–2.6) and cloacal MDT (39, 41, 42, 50, 59–109) in chickens*.

In Egypt, NDV infection results in variable mortalities and clinical findings. The clinical signs observed in commercial broiler chickens were severe depression, green diarrhea, paresis, and death within 48–72 h after the onset of the disease. Other signs including severe conjunctivitis, facial swelling, and birds standing dull with drooping wings were recorded in many studies. Besides, a drop in egg production reached 50% in layer flocks. Necropsies revealed congestion of the meningeal blood vessels and signs of septicemia in the form of congested subcutaneous (SC) blood vessels; congestion of the liver, spleen, and lungs; and gallbladder enlargement. Tracheitis and airsacculitis were seen in the respiratory tract. Hemorrhages on the tips of the proventriculus gland, greenish mucous content in the gastrointestinal tract, elliptical raised ulcers in the intestine, and enlarged hemorrhagic cecal tonsils were also observed (40, 110, 111). Variable mortalities (10–100%) were recorded in backyard and commercial vaccinated and non-vaccinated broiler poultry flocks (9, 49, 111–113). The clinical signs were respiratory distress and elevated mortality with nervous manifestation and deaths occurring with 24–48 h after the onset of clinical signs (48).

National efforts to update the knowledge about NDV/AIV prevalence and an active surveillance undertaken on 195 broilers and layers farms from 18 Egyptian provinces resulted in 41/195 (21%) positive for matrix gene of NDV and 24/195 (12%) positive for virulent NDV (44). During 2014–2015, the NDV genotype VII was reported with a percentage of 37.8% (114). There was a similar incidence of 37.5% in chicken flocks of 10- to 240-day-olds located in different districts of Sharkia province (115). Lower prevalence (12.5–16.2%) was recorded (49, 52, 116). A higher incidence of NDV with a percentage of 57.5% was detected during 2012–2015 (46) and 45.46% during 2019 (117). The highest incidence of NDV with a percentage of 86.2% was recorded in commercial chicken flocks during 2012–2015 (48). Moreover, Moharam et al. (9) screened 26 chicken flocks (backyard and commercial) during 2015–2016. Small-scale holders including farms keeping layers (*n* = 9), broilers (*n* = 4), or Balady chickens (*n* = 3) situated in provinces Beheira and Monufia of the Nile delta region. Commercial broiler farms were located in the provinces of Giza (*n* = 9) and Monufia (*n* = 1). Although all flocks were ND vaccinated, a virus was in both holders with a percentage of 84.6%. However, virulent NDV was detected in 30.76%. A recent study was conducted on 120 poultry flocks from 10 Egyptian provinces in the Egyptian Delta region during 2015–2019. The highest prevalence of virulent NDV was reported in broiler flocks (41.1%; 37/90), followed by layer flocks (38.4% 5/13). Baladi and Sasso chickens represented a confirmed NDV of 71.4% (5/7) and 33.3% (1/3), respectively (10).

**Figure 2:**
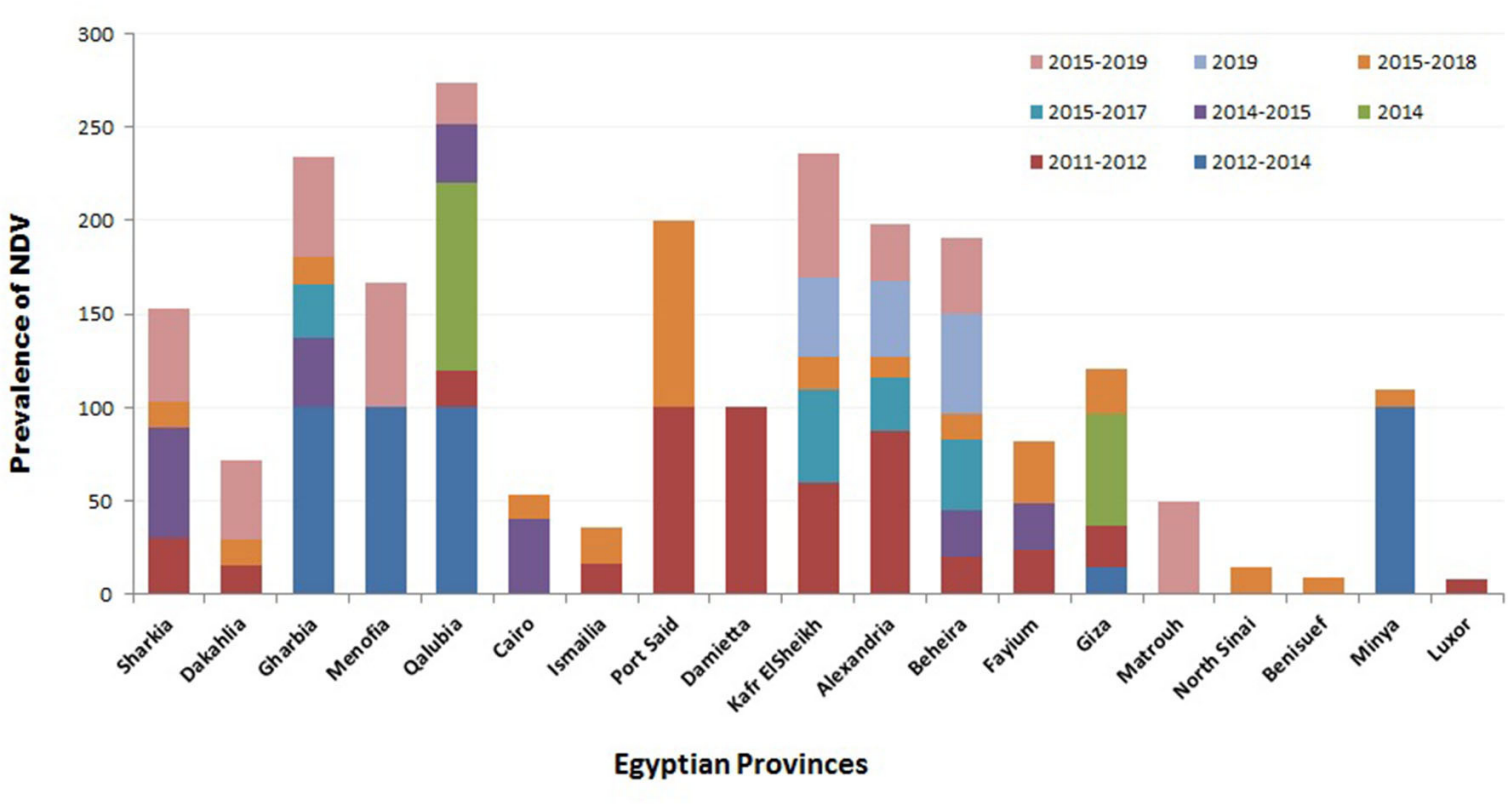
Summary of Newcastle disease virus (NDV) prevalence studies in Egypt according to geographical (Egyptian province) and time (year of detection) panels.

Regarding the prevalence of NDV according to localities, several studies were conducted to investigate the geographic prevalence of NDV in different provinces in Egypt. The overall incidence of NDV in Egyptian provinces during 2012–2019 (Figure 2) ranged from 8.3% in Luxor to 100% in many regions: Port Said, Damietta, Gharbia, Menofia, Qalubia, and Minya (44, 46, 47, 52). The incidence of NDV in Sharkia ranged from 14% (52) to 50% (10). No NDV was detected in some provinces: Qaluibia, Menofia, Matrouh, Qena (52), Giza (10), and Aswan (44). The variations mentioned above in the prevalence of NDV along the Egyptian provinces could be attributed to (i) individual concept of NDV surveillance either in domesticated or wild, free-living, and migratory birds; (ii) difference of testing procedures and sample size; (iii) variable distribution of poultry farm population and levels of biosafety and biosecurity; (iv) absence of efficient and reliable surveillance systems that are needed to document the disease status of a population at a given time; and (v) different factors like the disease awareness of persons reporting suspect cases. In this framework, and according the OIE, any national surveillance scheme for an animal/avian infection may be constructed on two different surveillance approaches: active as the regular periodic samples' collection by veterinary health authorities and passive surveillance, which is distinct from active surveillance, as birds are only tested if they show clinical signs, and then they are detected and reported to the national authorities (118). Besides mandatory standard parameters of ND prevalence, studies must be approved and applied.

### Migratory and Free Living Birds

Columbiformes birds including pigeons and doves can be infected with NDV; however, the disease in pigeons is mainly caused by pigeon-specific viruses, e.g., PPMV-1. Pigeons and doves had been implicated as amplification or reservoir hosts, as they were frequently infected with virulent strains of NDV (119, 120). Since 1981, the clinical pictures in pigeons were consistent with ND, and then the virus infection was serologically confirmed in diseased pigeons along the Nile delta provinces in 1984 (26). Compulsory vaccination of racing pigeons is a local act in some countries. However, control of NDV in wild pigeons is almost impossible (121). Thus, pigeons are considered threatening carriers for the poultry industry (122). Pigeons in Egypt are not regularly vaccinated. As described by Mansour et al. (123), Elgendy et al. (124), and Rohaim et al. (54), pigeons suffered from NDV and AIV infections. In previous records of pigeons, the NDV infection caused a variable range of mortalities and morbidities (125, 126). Abou Hashem (127) did isolate 24 antigenically similar PPMV-1 viruses from pigeons in the Egyptian provinces of Sharkia (*n* = 14) and Dakalia (*n* = 10). Experimental IV or intraocular (IO) inoculation of pigeons revealed diarrhea and nervous signs [3–11 days post-infection (dpi)] followed by 100% death. The virus was transferred by contact with birds and caused mortality of 60–80%. The prevalence of PPMV-1 in Dakhlia province showed 18/100 pigeons suffering from diarrhea and nervous signs. When pigeons were infected experimentally with field viruses, the re-isolation of PPMV-1 was up to 13 days pi; and using HI, the relationship between PPMV-1 and the LaSota strain was confirmed (128). Moreover, Amer et al. (129) explored the virulence and transmissibility of NDV experimentally in pigeons, as 5% of the infected birds showed greenish diarrhea and 95% had positive virus isolation, with a detection rate of 70% in contact pigeons. In Kafr El-Sheikh province during 2014, wild pigeons showed dullness, lethargy, and neurological signs in addition to petechial hemorrhages in the heart and brain, congested lungs and liver, and enlargement of spleen upon necropsy. Molecularly, the detected virus (Pigeon/Egypt/VRLCU/2014) belonged to genotype VI, a well-known lineage representing PPMV-1 in pigeons. Biologically, as shown in Table 1, the virus had ICPI and MDT of 1.2 and 86 h, respectively (54). In 2014, a pooled brain sample from three diseased free-living pigeons (torticollis and whitish green diarrhea) in Desouk, Kafr El-Shiekh province, Egypt, was identified by HI and partial F-gene sequencing as genotype VI (130). One study focused on the outbreaks of influenza and paramyxovirus co-infections among clinically diseased pigeons (7), where mortalities varied from 10 to 92.5% in both single and mixed virus infections, with an indicative clinical picture of PPMV-1 and/or AIV, as pigeons showed tremors (83.3%), torticollis (17.7%), wing and leg paralysis (25%), and greenish diarrhea. Also, respiratory signs were observed only in few naturally infected pigeons. NDV was detected in 67.8% of diseased pigeons, mostly in pigeons aged ≤ 1 month. Mesogenic NDVs are rarely traced in prevalence along the history of ND. Nevertheless, Hamouda et al. (131) utilized restriction fragment length polymorphism (RFLP) to identify nine strains (mesogenic/lentogenic PPMV-1); 27 (mesogenic/lentogenic NDV) in the Sharkia province, in addition to one velogenic strain for each NDV and PPMV-1, were also detected. In another study, brain samples were collected from 12 pigeon farms with severe neurological symptoms and greenish diarrhea and showed a mortality rate of 3.3–38.5%. The ICPI values were 1.41–1.51, and MDT was 64–69 h (Table 1). However, IVPI in chickens was zero, indicative of moderate virulence (mesogenic nature) in chicken. Phylogenetically, all tested viruses were in genotype VI (56). Viruses of genotype VI had also been circulating in apparently healthy pigeons (132, 133), and in the study of Sabra et al. (55), it was confirmed that at least sub-genotype VIg is probably maintained in healthy captive pigeons in Egypt, with an ICPI value of 1.31 (Pigeon/Egypt/Giza/11/2015).

To follow the transmission dynamics of avian avulavirus (velogenic viscerotropic ND-genotype VII), intranasally (IN) or intramuscularly (IM) infected 8-week-old non-vaccinated native Egyptian Balady pigeons were kept in contact with non-vaccinated commercial Arbor Acres broiler chickens (4 weeks of age). The IM-infected birds had 100% mortality for chickens and 53.3% for pigeons, whereas the mortalities of IN-infected birds were 70 and 6.6% for chickens and pigeons, respectively. The viral shedding was higher in the oropharynx compared with the cloaca for both IN- and IM-infected pigeons. The IN-infected pigeons continued shedding the virus from the oropharynx at 4–16 dpi, while IM-infected pigeons had no oropharyngeal shedding at 11 dpi. Contact chickens had typical ND clinical picture, with mortalities of 40–60% and with higher virus shedding titers upon contact with IM-infected pigeons compared with IN-infected ones (134). It is worth mentioning that PPMV-1 strains could be isolated from the intestinal tract of infected pigeons suffering from greenish diarrhea. Those infected pigeons enhance the spread of the disease due to potential exposure of other birds to contaminated food with the pigeon fecal material (121, 135). Doves are common birds that come into contact with chickens in Egypt, on either open rural farms or live bird markets (LBMs), transmitting NDV to other bird species. When doves were inoculated with NDV using several routes, they were highly susceptible and showed nervous manifestations and congested organs. They can also shed the virus and transmit it to contact-susceptible chickens. The velogenic viscerotropic NDV strain had been detected in cloacal swabs (15/140) from free-flying doves in different localities in Egypt (136). During 2014–2015, NDV was detected in doves and characterized as genotype VII from samples collected from Gharbia (NDV/Dove/Bassioun/Egypt/MS2/2014KR082486) and Kafr El-Sheikh (NDV/dove/Desouk/Egypt/MS5/2015KT006286).

Taken together, pigeons and/or doves play significant roles in introduction, maintenance, transmission, epidemiology, and distribution of emergent NDV viruses in Egypt. It is of high value to track pigeons, at least those in close vicinity to poultry farms. The disease caused by NDVs of pigeon origin varies according to several factors, including host, environmental, and co-infection scenarios as well as lack of hygienic precautions.

Quails had been introduced to the commercial poultry sector in Egypt mainly for food consumption and were considered carriers and/or susceptible hosts to NDV (58, 137, 138). In Egypt, El-Zanaty and Abd El-Motelib (139) isolated the viscerotropic velogenic ND from quails of the Assiut province. In the Suez Canal University, a farm of 5,000 quails had 1.6% mortality; preliminary diagnosis suggested ND. Diseased birds, 3 weeks old, showed mild respiratory and nervous signs. Dead ones showed focal hemorrhagic lesions in the respiratory system and hemorrhagic spots on the liver, spleen, kidneys, and heart, without any obvious lesions in the digestive tract. Seroconversion confirmed NDV antibodies (1:32–1:256). The NDV was successfully isolated with a percentage of 75%. Hemagglutination assay (HA) titers ranged from 1:8 to 1:2,048, while MDT/minimal lethal dose (MLD) of three strains was 70, 80, and 75 h (mesogenic). The experimental ND infection resulted in 25% deaths after 1 week (58). Aly (140) investigated PPMV-1 isolates experimentally in quails and revealed that it could cause mild infection with 5% mortality in quails, but contact pigeons displayed greenish diarrhea and nervous signs (25%) followed by deaths (20%).

Japanese quails in Egypt were also susceptible to infection with NDV genotype VII, where the virus caused 33% mortality in quails and 100% mortality in chickens, with a typical ND picture, which was more severe in chickens compared with quails (141). The low death rates accompanied with nervous involvement and different shedding patterns are shared observations in partially resistant birds such as pigeons (142), cormorants (143), and ducks (144) upon infection with virulent NDV viruses. Also, vaccination protected quails against NDV infection (145, 146).

Migratory and non-migratory free-flying wild birds can play significant roles in NDV potentiation, transmission, and spread. It is believed that most wild birds got NDV as a direct result of spillover from domestic poultry species (24). Few exceptions are noticed, as NDVs are endemic in migratory birds, such as PPMV-1, which had taken wild pigeons as a reservoir/adaptation host, which lead to (i) emergence of highly virulent forms of ND in other avian species but not pigeons, (ii) global dissemination of such viruses, and (iii) increasing threats to the commercial poultry sector (23).

In 1976, 9/386 NDV isolates were identified from cloacal swabs of migratory birds in Northern Egypt (Bahig, Burg El-Arab, and Ikingi Mariut) (147). The MDT in chicken embryos was 59.2–77.6 h. But the pathogenicity indices in pigeons were 0.14–1.98 and 1.95–3.02 for IM and IC applications, respectively. Intracloacal application showed that all isolates are lethal to susceptible chickens, which suffered from provoked neurological signs and intestinal lesions (velogenic features of NDV) (57). Most recently, Rohaim et al. (148) isolated and identified vaccinal APMV-1 (5/297 oral and cloacal swabs) during a survey of apparently healthy wild birds in eight Egyptian provinces during early 2014 to late 2015. The APMV-1 isolate from teal (NDV/Teal/VRLCU-EG/2015) had an MDT of 96 h and an ICPI of 0.4375, harbored the GRQGRL motif at its F protein cleavage site, and belonged phylogenetically to genotype II (100% identity with the LaSota vaccinal strain), which collectively indicate its lentogenic nature and highlight the potential reverse spillover of NDV live vaccines from domestic poultry to wild birds (148). Furthermore, El Naggar et al. (59) also characterized NDV (4/112) of wild bird origin in Egypt. Teal (*n* = 2), quail (*n* = 1), and cattle egret (*n* = 1) tested positive for NDV, whereas house sparrow samples were negative. The ICPI and MDT ranged at 1.6–1.83 and 63.2–65 h, respectively (Table 1), suggestive of the velogenic potential of the four isolates. They were molecularly clustered into VII genotype and had the RRQKRF polybasic motif at the F protein cleavage site. Under experimental conditions, the previously mentioned four isolates did cause a pantropic infection in chickens, and the LaSota-vaccinated one failed to survive the disease (59).

### Ducks/Aquatic Birds

Waterfowls, including ducks and geese, are less susceptible to NDV infection (24), as many NDV strains of different virulence had been isolated from either diseased or clinically healthy ducks, which raise the question of whether ducks/geese are only natural reservoirs or susceptible host to NDV (60, 61). Thus, the interest on natural infection of those birds with NDV has greatly increased (62–65). Prolonged viral shedding of waterfowl increased the risks of NDV of waterfowl origin (66). In Egypt, IM inoculation of Muscovy ducks with NDV genotype VII leads to only 5% mortality accompanied with higher and prolonged cloacal shedding compared with tracheal one. In contrast, IN inoculation did not cause deaths in ducks but elevated the tracheal shedding. The contact chicken had severe symptoms with very high mortality rates, emphasizing the fact that ducks are effective carriers of NDV (67). Besides, a virulent NDV of genotype VII was identified in 2/6 duck farms during 2017–2018 (68). Unexpectedly, NDV was also seen in co-infection reports, including two duck farms, but consistent with worldwide NDV reports from ducks (1, 64, 69). The role of ducks as a carrier of virulent NDV in Egypt remains to be investigated (68).

## Mixed Newcastle Disease Virus Infection

Mixed (co)-infection is defined as simultaneous infection of the same host with two different pathogens in the same time frame, a common event that is frequently occurring among birds, especially in intensive rearing systems and/or in developing countries. Several factors can control the outcome of co-infection, which could be either synergistic or antagonistic. This includes the interaction time, host immunity, and environmental conditions as well as biological activities of infectious agents. Viral–bacterial or viral–viral co-infection is highly common under poor biosecurity levels, which are frequently observed in Egypt. On the other side, viral interference is defined as a phenomenon in which initial virus infection prevents secondary homologous or heterologous virus by (i) blocking cellular receptors, (ii) competition on metabolic products required for viral replication, and (iii) initial sensitization of the host through virus-induced immune responses. Viral interference could interfere with proper diagnosis, as it could lead to undetectable/very low virus titers and atypical pathognomonic lesions (70, 71).

In Egyptian commercial poultry flocks, the disease outbreaks have increased during the last decade with high moralities and variable clinical pictures, especially respiratory signs. Lentogenic/velogenic NDV and high-pathogenicity AIV/low-pathogenicity AIV (LPAIV/HPAIV) are frequently reported from poultry, particularly in endemic areas, including Egypt. Both viruses share the same primary replication sites in the upper respiratory tracts of birds. Accordingly, both viruses could enhance or worsen the outcome of infection. Besides, co-infections with IBV were reported and represented significant alterations in the clinical picture, severity, and mortality rates (4, 72). Notably, a triple avian influenza subtypes (H5N1, H9N2, and H5N8) co-infection was also detected (68).

**Figure 3:**
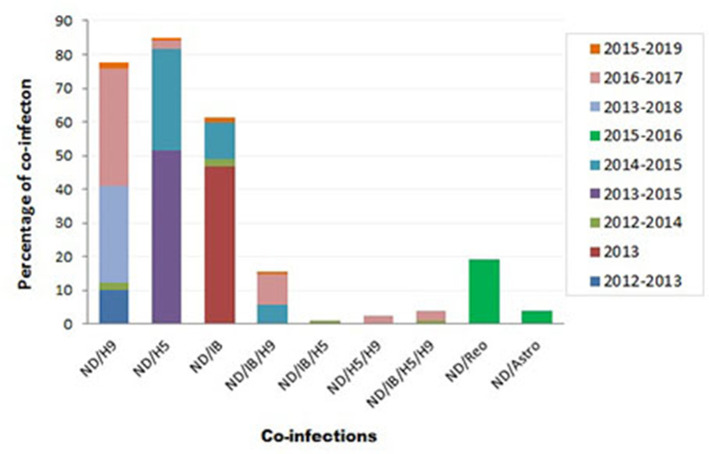
Summary of Newcastle disease virus (NDV) co-infection records with different infectious agents in Egypt.

Variable NDV co-infection with other viruses was recorded (Figure 3). In Sharkia province, concurrent infection of NDV/H9 was detected in 5/50 flocks (10%) through 2012–2013 (73), 1/42 flocks (2.4%) during 2012–2014 (74), and 2/7 samples (28.6%) during 2013–2018 (75). Thirty percent of mixed infection of NDV/H5 was recorded in 2014–2015 (115).

During 2012–2014, mixed infection of NDV with IB (2.3%), NDV/IB/H5 (1.2%), and NDV/IB/H5/H9 (1.2%) was recorded (4). Sooner, increased rates of NDV/IB co-infection (10.8%) and NDV/IB/H9 (5.8%) were reported during 2014–2015 (116). Interestingly, Zaher and Girh (76) reported higher incidence of NDV/IBV co-infection with a percentage of 46.67%. In broiler and layer chickens, the co-infection was 48.1% (38/79) and 45% (32/71), respectively. In pigeons, NDV/H5 co-infection (51.6%) was recorded during 2013–2015. The co-infections appeared to predominate especially with foreign breeds of pigeons (63.6%), between 1 month and 1 year of age (55%), in summer (71.4%) and 60% in the closed rearing system (7).

During 2016–2017, respiratory viral pathogens were screened among poultry flocks (*n* = 50), commercial broilers (*n* = 39), commercial layers (*n* = 11), quails (*n* = 4), Bluebird (*Sialis, n* = 1), and Greenfinch (*Chloris chloris, n* = 1). Results revealed that the incidence of single ND infection was 33.3%. Concurrent infection of NDV/H9N2 was reported with a rate of 7.7% in commercial broilers and 27.3% in layers. Other mixed infections, i.e., NDV/H5N1, ND/H5N1/H9N2, and ND/IB/H5N1/H9N2, were also observed (2.6% each). Mixed ND/IB/H9N2 infection was observed in three separate farms (9.1% each) (8).

At the period of 2017–2018, poultry flocks (*n* = 39) showed severe respiratory signs: 32 chicken flocks (19 broiler, seven native broiler, four commercial layer, and two breeder farms), six duck farms, and one flock of outbred turkeys. Out of 39 farms, nine samples were positive for vNDV. In chicken farms, various virus combinations with NDV were detected: H5N1/H5N8/H9N2/IBV/NDV or H5N1/H5N8/H9N2/NDV or H5N8/H9N2/IBV/NDV, and H5N1/H9N2/IBV/NDV or H5N8/H9N2/NDV. Two duck farms revealed mixed infection of H5N1/H5N8/H9N2/NDV (68). More recently, the co-infection rate of NDV/H9N2 (2/120; 1.6%), NDV/H5N1 (1/120; 0.8%), NDV/IBV (2/120; 1.6%), and NDV/IBV/H9N2 (1/120; 0.8%) was recorded in 120 commercial farms or backyard houses in 10 Egyptian provinces during 2015–2019 (10).

Moreover, other mixed infections of NDV with avian astrovirus (3.8%) or avian reovirus (19.2%) were also identified (9). Earlier, in surveillance among more than 100 chicken flocks at Sharkia province in late 1980, mixed NDV with IB, reo, or pox viruses was recorded among examined flocks (77).

## Sequence, Phylogenetic, and Deduced Amino Acid Analysis

### Sequence and Phylogenetic Analysis

A total of 408 Egyptian NDV F protein strains were obtained from the GenBank to be included in our analysis (335 from chicken, 52 from pigeon, 17 from other avian species, and four of unknown origin). The last ones were considered to be of chicken origin. The official molecular NDV reports started in Egypt as early as 2005 (41). However, the National Center for Biotechnology Information (NCBI) database had other Egyptian NDV strains that are assumingly from 1976, mid-1990s, and early 2000s. Consistent with the second NDV outbreak in Egypt (VII), poultry researcher focused more on NDV starting from 2011, with maximum detection in 2012 and 2014–2016 (Figures 1A,B). All recorded sequences belonged to NDV class II.

However, only 136 had a complete F protein gene, as also most of the sequences came from NDV strains of chicken origin (*n* = 117), followed by pigeon (*n* = 14), and finally few sequences from teal (*n* = 3), quail (*n* = 1), and cattle egret (*n* = 1). A pilot phylogenetic tree was constructed based on the recommendations of Dimitrov et al. (17), from which these criteria were mainly included: (i) alignment using complete F protein nucleotide sequences, (ii) construction using maximum likelihood method using the general time-reversible (GTR) model with Gamma distribution (G), (iii) application of the model with 1,000 bootstrap replicates and values of ≥70 were indicated above the tree branches, and (iv) involvement of independent strains with no obvious epidemiologic link.

Chicken (broiler, layer, and breeder) is hugely involved in the poultry industry of Egypt, with an investment of nearly LE18 billion at that time of recording (78). Large industrial farms follow a strict vaccination program with intensive biosecurity. However, backyard/family farms do not usually vaccinate their birds and either treat their diseased ones symptomatically or submit them for slaughter. Such a discrepancy in NDV control strategies and weak governmental imposition of field regulations contribute significantly in disseminating the NDV. In this regard, following the molecular pattern of NDV in chicken is of great value.

**Figure 4:**
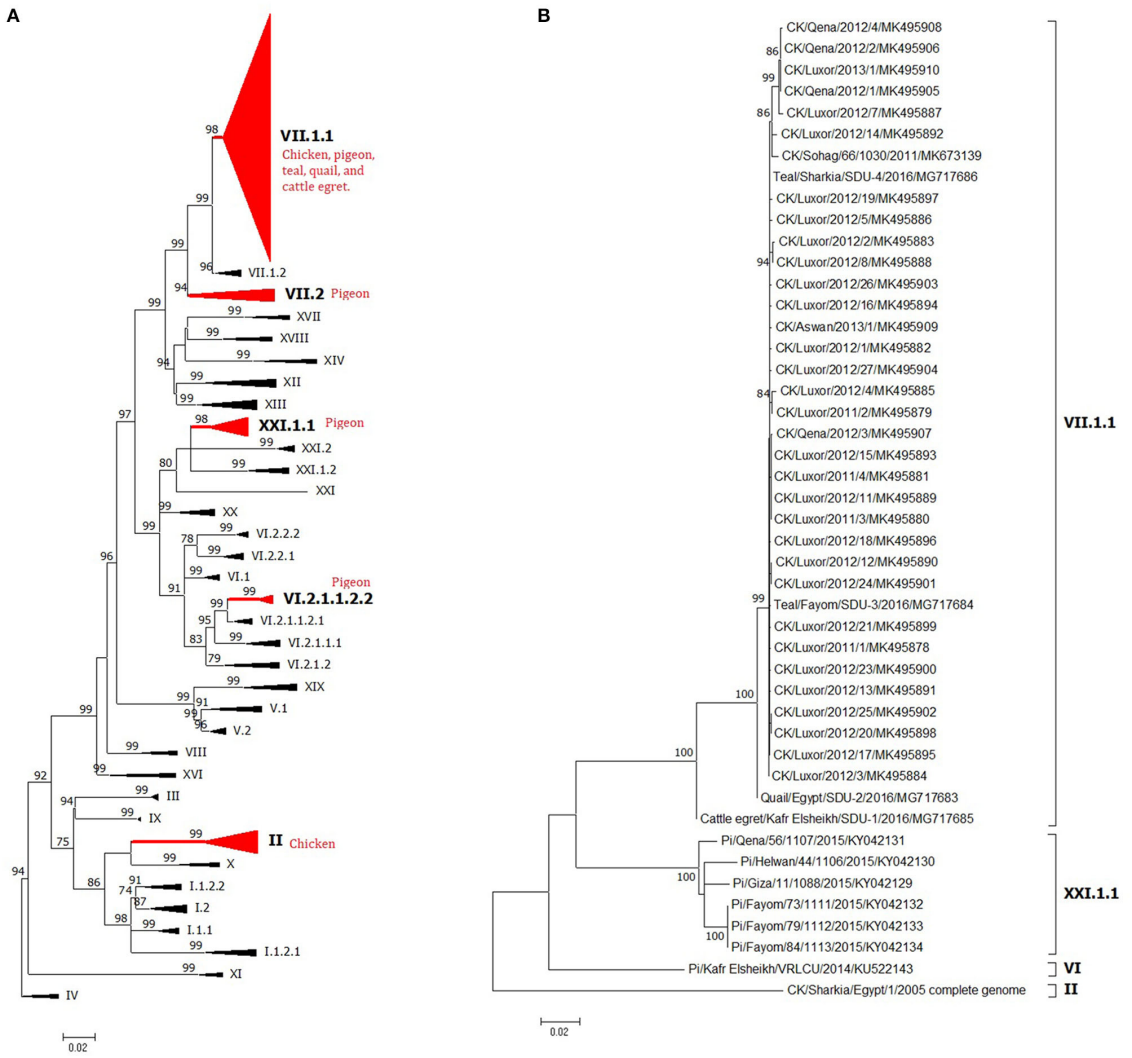
The phylogeny of Egyptian Newcastle disease viruses (NDVs) **(A)** full F protein gene or **(B)** full HN protein gene of different Egyptian isolates. The maximum likelihood (ML) tree was built based on the general time-reversible model with a discrete gamma distribution at 1,000 bootstrapping. All genotypes are in Newcastle disease virus (NDV) class II and were assigned with Roman numerals, as suggested by Dimitrov et al. (17). The taxons of F protein gene tree were compressed for better presentation. Sequences from Egypt are labeled with a red circle.

**Figure 5:**
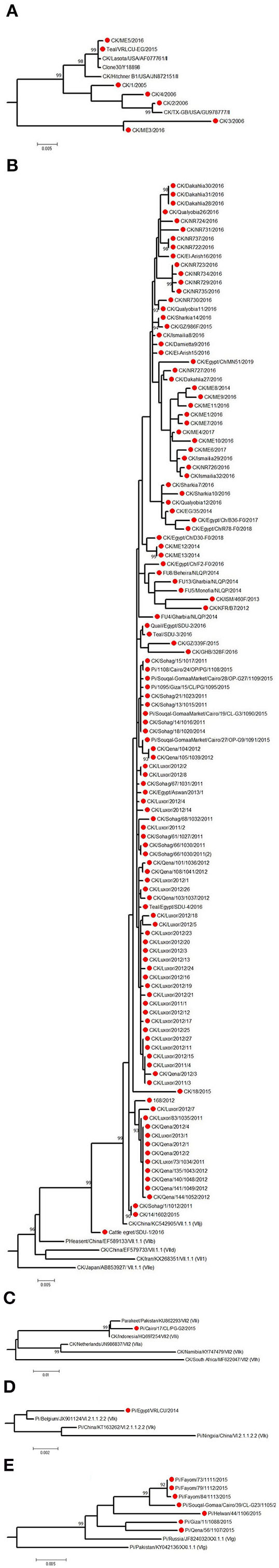
The detailed full F protein gene-based phylogenetic tree of Egyptian Newcastle disease viruses (NDVs), representing various detected strains from different bird species, which belonged to different lineages, as **(A)** genotype II, **(B)** genotype VII.1.1, **(C)** genotype VII.2, **(D)** genotype VI.2.1.1.2.2, and **(E)** XXI.1.1. The construction of the tree was done by using the maximum likelihood (ML) method, general time-reversible model, and bootstrap values of 1,000 (17). Sequences from Egypt are labeled with a red circle.

The NDV F sequences from chicken origin are grouped under the VII.1.1 or II genotypes. Genotype VII was reported from different parts of the world and was dominantly circulating in Egypt since 2011 (79) and was found across almost the whole country (Figures 1A,B). Previously, complete F strains were classified as VIIb (9) or VIId (6, 50). Recent literature started to follow the new classification system and predominantly described VII.1.1 from chickens (10, 51, 80). Some partial F sequences were identified as VIIj (81), or generally as VII (79, 111), and most surprisingly as genotype VI (39) or I (accession number KR535623). However, studied sequences were too short to be considered for conclusive genotypic taxonomy. Since the VII.1.1 included anyhow previously characterized members of VIIb, VIId, VIIe, VIIj, and VII1, we could assume that all previously speculated NDV VII strains from chicken are actually one genetic linage of viruses or one genotype known as VII.1.1, which is responsible for the second epizootic outbreak/wave of NDV in Egypt, and still endemic in Egypt since then (Figures 4A, 5B). Meanwhile, NDV genotype II from chicken was initially described in 2005 (41) and 2006 (42), when it was responsible of the first reported NDV outbreak in Egypt. Subsequently, it was displaced by genotype VII. Nevertheless, genotype II is still existing but to a lesser extent (51) (Figures 4A, 5A). Pigeons are brought in Egypt for several reasons, including passion, gaming, investment, and meat production. They are mostly raised in spindly wooden columns that look like a medieval siege tower. Unfortunately, pigeons are a high potential source of NDV spread at least to commercial/backyard poultry sector due to their migratory behavior, free-living system of rearing, close contact with other birds in or out LBMs, and reluctance of owners to vaccination. Despite being a carrier reservoir for NDV (134), NDV strains of chickens were more pathogenic and transmissible to chickens compared with those from pigeons (82). The NDV surveillance system in the pigeon is quite weak and does not reflect the actual situation.

The low number of NDV/pigeon paramyxoviruses detected in pigeons reflected a high level of genetic diversity. At first, Rohaim et al. (54) reported the PPMV-1 from a single case (one diseased pigeon; KU522142) in 2014 and was classified as VI (sub-genotype VIb). Consequently, two investigations in clinically affected pigeons recorded partial F protein sequences of PPMV-1. Mansour et al. (7) found three different NDV genotypes during 2013–2015 (Ia, II, and VI), where the VI sequences belonged to VIb.2, a newly emerged cluster that was described mainly in Europe, while KU522142 clustered with VIb.1/re, another classical pigeon NDV lineage from Europe. The same genotype (VIb.2) was also found in 2016 and was renamed as VIg (56). Complete F protein gene sequences of VIg were also defined in apparently healthy pigeons in 2015 (55). Based on the classification system of our study, complete F protein sequences from pigeons clustered into four groups: the newly proposed genotype XXI (XXI.1.1; *n* = 7), VI (*n* = 1), VII.1.1 (*n* = 5), and VII.2 (*n* = 1). The XXI.1.1 separated basically VIg strains from the VI genotype. The sequence, KU522142, remained in VI and assigned in the sub-genotype VI.2.1.1.2.2, while VII.1.1 and VII.2 were seen also in 2015 (83) and described in Figures 4A, 5B–E. Other strains that had a partial F protein gene (identified as genotypes I and II plus unidentified ones) need further confirmation/analysis following complete F protein gene sequencing.

Other birds, including wild ones, are considered as another introducing, disseminating, and maintaining factor of NDV in Egypt due to their migration or involvement in LBMs. They are also involved directly or indirectly in the wild–domestic–human interfaces, imposing the importance of continuous surveillance of pathogens. All five complete F protein gene sequences from quail (*n* = 1), Cattle egret (*n* = 1), and teal (*n* = 3) belonged to genotype VII.1.1 (59) except one sequence from teal (148), which was in genotype II (Figures 4A, 5A,B). Another 12 NDV partial F protein sequences from passerines (*n* = 1), dove (*n* = 2), house sparrow (*n* = 3), cattle egret (*n* = 2), white wagtail (*n* = 1), white-throated kingfisher (*n* = 1), and hoopoe (*n* = 1) were found in genotype VII, while ostrich (*n* = 1) was placed in genotype II.

For the HN protein, a total of 46 complete gene sequences were collected and involved in the phylogenetic tree (Figure 4B) to collect some evolutionary criteria for an important NDV surface protein in Egypt. Both Egyptian HN and F protein followed the same phylogenetic topology. They were found in genotypes VII.1.1 (strains from chicken mainly, teal *n* = 2, quail *n* = 1, and cattle egret *n* = 1), II (one sequence from chicken), and VI or XXI.1.1 (strains of pigeon origin).

### Deduced Amino Acid Analysis

The F protein cleavage site (112-117) is considered a major determinant factor that plays a role in virus pathogenicity/virulence (19). However, other factors, for example, the HN stem region and globular head, are also involved (12). All isolates of Egyptian NDVs had an F protein of 552aa length. The deduced aa analysis showed the relative stability of F protein cleavage site in the Egyptian NDV isolates overtime, which tend to be genotype-specific, not species-specific. NDV isolates in VII.1.1 and VII.2 (from chicken, pigeon, teal, quail, or cattle egret) had the velogenic motif RRQKRF except one chicken isolate that had the RRKKRF motif.

Genotype II strains had either the velogenic motif RRQKRF (in chicken and pigeon) or the lentogenic motif GRQGRL (in chicken and teal). Pigeon NDV strains in XXI.1.1 had the velogenic KRQKRF motif, while pigeon strains in genotype VI and I had the velogenic RRQKRF and lentogenic GKQGRL ones, respectively. Two strains from pigeon (MF614961) and chicken (MK604215) also had the lentogenic motifs GRQGRL and GKQGRL, respectively (unidentified genotype; not in Supplementary Tables 1, 2). The previously mentioned 12 NDV partial F protein sequences from wild birds had all RRQKRF residues at their cleavage site, except the ostrich isolate, which had the RNQGRL motif. As previously reported, aa changes in the cleavage site affected the fusion efficiency of the F protein (84), gradual dominance of virulent strains in quasispecies (85), and virulence of the virus (ICPI decreased in Q114R mutant viruses) as reported by Samal et al. (86). However, the clinical pathogenicity of the virus under natural conditions is usually influenced by other viral, host, and environmental factors. Interestingly, Nagy et al. (51) reported high pathogenicity indices in lentogenic motif harboring NDV isolates of chicken origin, emphasizing the importance of following the OIE recommendations in determining virulence regarding NDV of field origin.

The F protein signal peptide (1–31) was highly variable among the Egyptian strains, consistent with the findings of Orabi et al. (6). Other regions, including the fusion peptide (117–142); heptad repeats a, b, and c (HRa 143–185, HRb 268–299, and HRc 471–500); transmembrane (TM) domain (501–522); and cytoplasmic (CT) tail (523–553) were subjected to aa analysis, where several mutations were reported, which might affect the folding and fusion activities of the protein as described in the fusion peptide (87, 88) or the HRa, b, and c (89). The HRa is also supposed to include a potential antigenic epitope (90, 149–163); however, the Egyptian NDV isolates had only few reports of aa substitutions in this epitope (V168I and D170N). The TM domain affects the structural confirmation of the F protein, F–HN protein interaction, and fusion activity (91). Also, mutant CT domain modulates the F protein biological characters, virulence, and pathogenicity (92). D170N was reported in VII.2 and VII.1.1 (one pigeon and four chicken strains, respectively), which is a neutralizing epitope (93). Also, residues D479 and S486 are critical for the fusogenic activity (94) (Supplementary Tables 1, 2).

The HN protein of NDV is a surface glycoprotein that mediates several functions, including (i) attaching of NDV to cellular sialic acid receptors (95), (ii) promoting F protein fusion activity (96), (iii) facilitating the NDV budding by its neuraminidase (NA)-mediated receptor cleavage (97), and (iv) determining NDV tropism and virulence (98). Structurally, the NDV HN protein consists of CT tail, N-terminal TM domain, a stalk region, and a C-terminal globular head. The HN stalk forms an interaction with the F protein through a stretch of amino acids (aa positions 74–110) that forms two conserved heptad repeats (HRA and HRB) (99).

The HN protein length of Egyptian NDV isolates was 572aa except the parent NDV isolate (II) from 2005 (FJ939313), which was slightly longer (578aa). The NDV HN length affects the replication and biological properties of the virus as extended HN showed increased HA titer and receptor binding but impaired NA, fusion, and replication abilities. However, the virulence of virus was not changed (100). TM and stalk domains of the Egyptian NDV strains were highly conserved among genotypes (for example, VII.1.1, XXI.1, VI, and II), with few substitution mutations, which may affect the structure and activity of the HN protein (99, 101).

Major epitopes in the C-terminal head of HN were also investigated. As it is basically involved in antibody recognition (102), a single aa change in that major linear epitope ^345^PDKQDYQIR^353^ (1/1–4) allows the escape of its corresponding antigenic variant from neutralizing monoclonal antibodies, which was reported at least at position 347 (103, 104). It might explain the high virulence of chicken VII.1.1 isolates in chickens compared with others. Other antigenic epitopes within the HN protein were compared as shown in Supplementary Tables 3, 4.

Amino acid residues involved in receptor recognition (95), HAD ability, NA activity, fusion activity (105), interaction with F protein (106), head–stalk linker region (107), and predicted B-cell epitope (108) were highly conversed with few exceptions. Due to the intensive unplanned vaccination in Egypt, selection/vaccination pressure could explain the occurrence of such aa substitutions in the epitopes of Egyptian NDV HN overtime, which in turn may affect, to different levels, the vaccine efficacy, induced immunity, and virus shedding in birds as a result of antigenic variability (108). HN protein is a determinant viral factor for thermostability, as mutant NDV viruses (S315P and I369V) were more thermostable and possessed more HA titers and NA fusion activity (109).

## Vaccination Strategies and Challenges

Besides good biosecurity regimens, the control of ND principally accounted for mandatory preventive vaccination of flocks and hygienic culling of infected birds. In Egypt, many NDV commercial traditional/classical genotype II vaccines are used in the Egyptian poultry field such as (i) live seed virus vaccine strains of LaSota, Hitchner, VG/GA, clone 30, PHYLMV, and others or (ii) inactivated (killed) virus vaccines, mainly the LaSota one (153). Recently, several recombinant and novel inactivated (Genotype VII; GVII) vaccines were introduced gradually to cope with the continuous evolution and spread of velogenic NDV-GVII (154, 155). ND is endemic in Egypt, and there is an enormous pressure from the field circulation of diverse genotypes II, VI, and VII; massive poultry production; and direct and/or indirect contact with free-living and migratory birds, which generally represent a significant challenge to poultry holders. Currently applied vaccination strategies are relatively effective in preventing severe illness and death of infected birds but may fail to prevent infection or shedding of the virus. In Egypt, the main goal of scientists and poultry producers is to minimize the economic impact of NDV infections. Accordingly, innovative vaccination strategies were applied to potentially increase protection and reduce viral shedding, and presumably the spreading and the transmission of the virus. Here, we reviewed all studies related to development of ND vaccine technology and strategies. The extent to which heterologous vaccines can protect against different genetic variants of NDV is still controversial. The applied preventive programs in Egyptian provinces include live (genotype II) and inactivated vaccines (genotypes II/VII). Proper vaccines and the design of efficient vaccination programs shall give the best protection against clinical disease and prevent/reduce either mortality or virus shedding in vaccinated flocks.

Over the past 25 years, many vaccination practices were tested experimentally in Egypt (Supplementary Table 5). Some studies were designated to evaluate different vaccines and/or vaccine regimens applied in Egypt. Upon evaluation, the ND live virus vaccine protected from clinical disease (90%) and deaths (80%) in case of homologous challenge with GII of VG/GA live strain (77) and HB1 with LaSota live strains (156) but only 75% protection rate from clinical disease and deaths as reported by Lebdah et al. (157). These protection percentages were raised up to 100% upon inclusion of inactivated vaccines with HB1 and LaSota live vaccines (156, 157). However, the results of Mohamed et al. (158) were drastic when they used DNA vaccine containing F and HN antigens in one dose (30–40%); then they improved the protection to 90–100% by introducing two or three vaccine doses at 1 week apart.

In most broiler chicken farms, it is recommended to administer several doses of live attenuated vaccines that should be primed as early as possible (159, 160). This could begin directly after hatching (at 1 day old) with HB1, followed by a booster dose of LaSota or Clone 30 vaccine two times at 2 weeks apart. As a result of insufficient protection of live vaccines in some intensive rearing localities, inactivated vaccines are usually included at the seventh or 14th day of age to maximize the protection. Day 1 recombinant vaccine application is still of limited use (153, 161).

After the involvement of new velogenic genotype VII of NDV (79), many studies were performed to evaluate the existing regimens to protect against the circulating field viruses, develop vaccine preparation, and update virus vaccine seeds (155, 158, 162–165). Three schemes of vaccination were proposed; the first included a heterologous vaccine regimen (massive genotype II vaccines) against challenge velogenic NDV genotype VII. The use of multiple live vaccines (genotype II; HB1, LaSota, and clone 30) induced lower protection that ranged from 72 to 93.4% (159, 166). In addition, programs included both inactivated and live genotype II-based vaccines; 100% of birds were completely protected from mortality upon heterologous virus challenge with genotype VII (45, 167). However, Nemr (168) and Shahin et al. (160) recorded 93.66 and 96% protection, respectively. On the other hand, trials to use only one shot of inactivated genotype II vaccine was contradictory, as Sediek et al. (169) recorded extremely low protection (33.3–46.7%). Meanwhile, Kilany et al. (162) reported 80% protection against heterologous challenge. Regarding the tracheal and cloacal shedding, all aforementioned studies revealed variable quantitative (0.6–6.6 log base 10 and two higher challenge up to 8.5) positive shedding from the second day up to 10th day post-challenge with genotype VII–velogenic NDV (Supplementary Table 5), which establishes the field virus transmissibility particularly after heterologous vaccination and non-strict hygienic measures.

In an unprecedentedly swift response to develop and manufacture an anti-NDV–genotype VII vaccine, inactivated virus vaccine was prepared from currently circulating velogenic NDV genotype VII, national and regional companies and academic institutions are exploring the numerous strategies. Subsequently, the second vaccine scheme was anticipated to be based only on homologous vaccination tested experimentally since 2015. Regarding the use of one killed vaccine S/C in chickens containing GVII strains, the results revealed 100% protection against clinical signs and mortality post-challenge with a similar genotype (162, 164, 168), which was accompanied with reduced viral shedding to <2.2 till the 10th day post virus challenge. Other findings included the stoppage at the fifth day (165) or the complete absence of virus shedding (154). Nevertheless, Sedeik et al. (169) used an inactivated NDV genotype VII vaccine to immunize the birds against a challenged virus from the same genotype, where they showed protection of only 53.3% with presence of clinical signs and virus shedding. The low protection was explained by using an inactivated vaccine prepared from a Korean NDV strain (KBNP-C4152R2L strain, INC., Korea) that is different from the circulating NDV strains in Egypt, which was used as a challenge virus. Conversely, a mucosal inactivated vaccine containing genotype VII failed to protect when used once (0%), which increased to 60% upon booster/second vaccination and 100% when inactivated oil-based vaccine was applied with the mucosal one (163).

The Third Scheme was based on the conjunction of GII and GVII to get the benefit from a gift of naturally attenuated viruses of genotype II (Lentogenic vaccines) and broaden the scope of immunity by inclusion of killed genotype VII. The trials of Bastami et al. (170) of mixed use of different permutations from live vaccines, HB1 and LaSota with either native or foreign inactivated genotype VII to protect chickens against challenge with virulent GVII-NDV, had a range of 90–100%. The best trial gave 100% protection from deaths and the lowest shedding (22.2%) when primed at day 5 of age with simultaneous live HB1 and native inactivated and then two doses of live LaSota (10th and 21st days of age). Also, 100% protection with non-significance as low as less than one log10 was reported by Hassan et al. (167) when using Live LaSota (clone 79) (genotype II) in WOW (water in oil in water inactivated vaccine) (VIIj) against a virus challenge by velogenic ND (VIIj). In another study, even with more than five doses of both live (GII) and inactivated (GVII), the virus was detected in tracheal swabs of chickens through 3–5 days post-challenge with velogenic NDV genotype VII (159). This establishes that not only the diversity of vaccine and field viruses or number of doses but also efficient administration, dose, and other factors may limit the scheme's efficacy. In a different study, both live LaSota and inactivated genotype VII could not fully protect against challenge with velogenic genotype VII from teal (NDV/Teal/Egypt or egret, and NDV/Cattle egret/Egypt; 60 and 40%, respectively). However, the virus was shed from challenged chicken (4–6 dpi), which set the need for more epidemiological investigations of the impact of non-chicken strains of NDV genotype VII (59).

Concerning PPMV-1, limited investigations (Supplementary Table 6) were set to evaluate the vaccine strategies against the virus. Studies concluded that complete protection could be achieved using a homologous inactivated vaccine with good adjuvant to be administered either SC or IM. Amer (171) prepared inactivated cell culture PPMV-1, which induced HI antibodies (GMT: 64) on the third week post-vaccination. Amer et al. (172), Khedr et al. (173), and Soliman et al. (174) succeeded in protecting 100% of challenged pigeons using inactivated PPMV-1 vaccine. Combined live HB1 or LaSota vaccines with inactivated PPMV-1 gave high protection (100%). It was proved that both virulent NDV and PPMV-1 circulate among pigeons in the Sharkia province of Egypt (131), necessitating the review of vaccine strategy of both viruses at risk.

Despite the diversity of virulent genotypes reported along the recent history of the disease in Egypt, all NDV strains are clustered into one serotype. This explains that under laboratory conditions, a vaccine prepared from any strain or genotype can induce antibodies to protect birds (decrease/prevent the clinical signs and deaths) against challenge with the virulent viruses (149–152, 175). However, other components such as cellular immunity should be taken into consideration, as it is not defined by serotype and could not be achieved by using inactivated vaccines, which is an available option for genotype VII.

Successful protection against circulating diverse NDVs in Egypt is a multifactorial issue. Under experimental and some field vaccination programs containing NDV genotype II, the vaccine could provide good protection (96–100%) against infection with heterologous Newcastle viral strain (genotype VII) and reducing the amount of viral shedding (45, 155, 159, 160, 176). However, upon field conditions, multiple factors could contribute to a considerable reduction in the effectiveness of vaccination, including (i) the frequent incidence of NDV infection, even in vaccinated birds, as being vaccinated does not prevent infection or virus shedding; (ii) improper vaccination; (iii) immune suppression of infectious or non-infectious origin; (iv) faulty program that may cause loss of cell-mediated immunity; and (v) prejudging viral mutation/changes in the genomic sequence of the virus, which can lead to presence of many serological variants (3, 159). Also, other factors include insufficient biosecurity procedures and the probable threats for disease transmission from wild/migratory birds to domesticated birds and vice versa. Strict and improved biosecurity must be at the solution's core to minimize the environmental virus load and halt its mutation. Contingent to the presented data, it is essential to have a more detailed analysis of the biological and antigenic characters of currently circulating NDV strains and the efficacy of commonly used NDV vaccines for protection against the NDV isolates in a frame of national plan utilizing the accumulated knowledge and unifying the system of investigation for better controlling the NDV in Egypt.

## Conclusion

ND is a highly prevalent viral disease, which is caused by various strains of NDV. The disease has potential intercontinental endemicity that further leads to economic crises due to high morbidity and mortality, long-lasting limitations on international trading operations, and increased costs of veterinary management. As a result of its extensive spread, countries like Egypt is trying to manage effective preventive and control measures, which preferably includes (i) culling and slaughter, (ii) active surveillance in domestic and wild birds, (iii) intensive biosafety measures, and (iv) strong vaccination programs. In Egypt, it is difficult to control the backyard/rural breeding of birds, which makes it relatively problematic to follow such regulations. Culling is not always an option due to the lost cost. The reliance on genetically dissimilar vaccines may not eliminate the persistent NDV viruses in commercial poultry sector, probably due to the presence of some antigenic variations. However, it is not the sole cause of vaccination unreliability in immunocompetent birds (177), particularly in several Egyptian studies in current commentary revealed protection with heterologous and mixed genotype with both live and inactivated vaccination (159). In accordance with field isolates, the usage of vaccine matched strains will probably help in extra control of NDV (178) with priming of live lentogenic strains to induce specific cell-mediated immunity, which could not be induced by a homologous one being inactivated virulent genotype VII. The NDV epidemiological gap in Egypt is still expanding with time, and accordingly, we advise the following recommendations for better outcomes:

(1) Following the OIE recommendations for identification of isolated NDV strains, which includes using at least two pathogenicity indices (ICPI and MDT at best), plus sequencing F protein cleavage site or monoclonal antibody HI matching using a reference panel (2). This shall avoid mischaracterization of the pathogenic nature (velogenic/mesogenic/lentogenic) of the isolated strains, especially with increased detection of lentogenic strains that possess a velogenic motif at their F protein cleavage site.
(2) For NDV isolates from birds rather than chicken, pathogenicity indices shall be performed in both actual bird of origin and chicken, as different indices may implicate the adaptation of the virus in both hosts, despite the close genetic relatedness (179).
(3) Full epidemiological data should be reported in future studies, including size of investigated flock, age of birds, the total number of raised birds, morbidity and mortality, the scientific name of bird involved, type of production, history of vaccination, and province (geographical site) in Egypt.
(4) We also advise to use the criteria proposed by Dimitrov et al. (17), particularly regarding full F protein sequencing and epidemiological parameters. Partial F protein sequence misleads accurate characterization, in either phylogenetics or detection of emergent and/or antigenic escape mutant NDVs. When possible, also the HN protein should be fully sequenced.
(5) We encourage broader observations of NDV viruses in free-ranging, aquatic, and migratory birds in Egypt and also not to ignore the possibility of viral or bacterial mixed (co)-infection in flocks under study.
(6) Application of biosecurity is a compulsory solution since vaccination alone cannot completely prevent field virus shedding.
(7) When applied, we advise to prime live lentogenic (genotype II) with a subsequent inactivated (homologous or heterologous) vaccine to achieve specific cell-mediated and humoral immunity and to broaden the scope of protection.

## Author Contributions

AAE, SM, and FM: conceptualization. SM, RE, FM, and AAE: data curation. SM, RE, FM, and AAE: formal analysis. SM, RE, FM, ARE, EH, MA, AAE, HA, and MI: investigation. AAE, ARE, and SM: supervision. SM, RE, and FM: writing—original draft. SM, RE, FM, EH, MA, MI, HA, AAE, and ARE: writing—review and editing. All authors contributed to the article and approved the submitted version.

## Conflict of Interest

The authors declare that the research was conducted in the absence of any commercial or financial relationships that could be construed as a potential conflict of interest.

## Supplementary Material

Supplementary Table 1 Fusion protein analysis in Pigeons.

Supplementary Table 2 Fusion protein analysis in chickens and other birds.

Supplementary Table 3 Functional domains and epitopes in HN of Egyptian NDV strains.

Supplementary Table 4 Head-stalk linker region involved in fusion triggering.

Supplementary Table 5 Vaccination strategies in chickens (NDV).

Supplementary Table 6 Vaccination strategies in pigeons (PPMV-1).
