# Supplementary material for: Avian Paramyxovirus Type 1 in Egypt: Epidemiology, Evolutionary Perspective, and Vaccine Approach
Source: Front Vet Sci. 2021 Jul 15;8:647462. doi: 10.3389/fvets.2021.647462 (PMC8320000; doi:10.3389/fvets.2021.647462)
Supplement: Supplementary Table 1 — Fusion protein analysis in Pigeons. [file Data_Sheet_1.PDF]

Table S1: Fusion protein analysis in Pigeons:

Species	Genotype	Number of strains	Cleavage site (112-117)	Fusion peptide (117-142)	HRa (143-185)	HRb (268-299)	HRc (471-500)	Transmembrane domain (501-522)	Cytoplasmic tail (523-553)
**Pigeon**	**XXI.1.1**	7	KRQKRF				S486G, V491I (one strain)		T550A, T551A (one strain)
	**VII.1.1**	5	RRQKRF	I121V, S132A	N145K, A176S	H272Y, Q279H, N296Y (one strain)	D489E, K494R	V506I (one strain), V513I, F514S, I520A	I537L, T553A
	**VII.2**	1	RRQKRF	I121V, S132A	D170N, A176S	H272Y, V287I	R480K, A482T, K494R	V506A, V513F, A516V, I520G	I537L, T553A
	**VI**	1	RRQKRF		V179I	H272N, N294Y		V506I, V509I, I520V	I537L,
	**II**	1	RRQKRF		Incomplete				
	**I**	15	GKQGRL	F117L, S124G, S132A, A139S	Incomplete				

Fusion peptide FIGAIIGSVALGVATSAQITAAAALI

HRa QANQNAANILRLKESIAATNEAVHEVTDGLSQLAVAVGKMQQF

HRb LITGHPILYDSQTQLLGIQVNLPSVGNLNNMR

HRc NNSISNALDRLAESNSKLDKVNVKLTSTSA

TMD domain LITYIVLTVISLVFGALSLILA

CT domain CYLMYKQKAQQKTLIWLGNNTLDQMRATTRT

Table S2: Fusion protein analysis in chickens and other birds

Species	Genotype	Number of strains	Cleavage site (112-117)	Fusion peptide (117-142)	HRa (143-185)	HRb (268-299)	HRc (471-500)	Transmembrane domain (501-522)	Cytoplasmic tail (523-553)
**Chicken**	**VII.1.1**	118	RRQKRF/ RRKKRF (one strain)	V121L, V121I, S124G, A126V, G128E, V129F, A130S, T136A (only in one strain)	N150S/K, I151T/L, L152H, L154F, V168I, D170N	L268R, N296Y (one strain)	D479G, N485S, K487R (one strain), E489K, N492K (one strain), S499P	I505M, F514S/C, L517F	K530T, L539F, **N541D**, N542I, Q546H, T550P (only in one strain), T551M
	**II**	4	RRQKRF	V121I, S124G/R, Q134L (one strain)	Q143P (one strain), S176A	Y272N, H279Q, N288T, L295I (one strain)	D479N, R480K, A482E, S486R (one strain), E489D, R494K	V509I, I510T, I513V, A516I, A520V (one strain)	T550A, R552K, A553M
		2	GRQGRL	F117L, V121I, S124G	Q143P (one strain), S176A	Y272N, H279Q, N288T	D479N, R480K, A482E, S486R (one strain), E489D, R494K	V509I, I510T (one strain), I513V, A516I, A520I (one strain)	T550A (one strain), R552K, A553M
**Quail**	**VII.1.1**	1	RRQKRF						
**Cattle egret**	**VII.1.1**	1	RRQKRF						
**Teal**	**VII.1.1**	2	RRQKRF						
	**II**	1	GRQGRL	F117L, V121I, S124G	S176A	Y272N, H279Q, N288T	D479N, R480K, A482E, S486R, E489D, R494K	V509I, I513V, A516I, A520I	R552K, A553M

**Fusion peptide**	FIGAVIGSVALGVATAAQITAAAALI
**HRa**	QAKQNAANILRLKESIAATNEAVHEVTDGLSQLSVAVGKMQQF
**HRb**	LITGYPILYDSHTQLLGIQVNLPSVGNLNNMR
**HRc**	NNSISNALDRLAESNSKLEKVNVRLTSTSA
**TMD domain**	LITYIVLTVISLIFGALSLALA
**CT domain**	CYLMYKQKAQQKTLLWLGNNTLDQMRATTRA

Other birds had the velogenic motif RRQKRF except ostrich RNQGRL

Antigenic epitopes in CK, Pi, other birds: (Chen et al., 2001)

D72, E74 (except one D74 one VII.1.1), **A75**, R78 (except K78 in VII.1.1, II+ II teal, and VI, VII.2 pigeon), **A79**, S157, T161, **D170** (except 4 strains VII.1.1 CK), G171, L343, A378
