# Supplementary material for: Avian Paramyxovirus Type 1 in Egypt: Epidemiology, Evolutionary Perspective, and Vaccine Approach
Source: Front Vet Sci. 2021 Jul 15;8:647462. doi: 10.3389/fvets.2021.647462 (PMC8320000; doi:10.3389/fvets.2021.647462)
Supplement: Supplementary Table 3 — Functional domains and epitopes in HN of Egyptian NDV strains. [file Data_Sheet_2.PDF]

Table S3: Functional domains and epitopes in HN of Egyptian NDV strains

Species	Number of sequences included	Genotype	Year	Peptides			Pathogenicity indices	References/ notes
				TM (25-45)	Stalk domains			
					HRA (74-88)	HRB (96-110)		
**Chicken**	1	II	2005	Consensus of FRIAILLLTVVTLATSVASLV	Consensus of LGSNQDVVDRIYKQV	Consensus of LLNTE**TT**IMNAITSL	**ICPI** 1.75, **MDT** 55h, **IVPI** 2.5 (velogenic).	Mohamed et al., 2009
**Chicken**	33	VII.1.1	2011-2013	Consensus of FRIAVLLL**MA**M**I**LAISAA**A**LA, where M33T (5 strains), A34T (4 strains), A34V (one strain) A43T (one strain). *Mostly in 2012, one in 2013.	Consensus of **LS**S**G**QDV**I**DRIYKQV Except L74F in one strain, Q78R in one strain	Same consensus in II except T101S and T102V		
**Quail**	1	VII.1.1	2016	Same consensus in VII.1.1	Same consensus in VII.1.1 except G77S	Same consensus in II except T101S and T102V	**ICPI** 1.6-1.83 **MDT** 63.2-64.1h (velogenic in chicken).	Failed protection in Lasota-vaccinated contact chicken (El Naggar et al., 2018).
**Teal**	2	VII.1.1	2016	Same consensus in VII.1.1	Same consensus in VII.1.1	Same consensus in II except T101S and T102I or T102V		
**Cattle egret**	1	VII.1.1	2016	Same consensus in VII.1.1 except A34V, I36T	Same consensus in VII.1.1 except G77S	Same consensus in II except T101S and T102I		
**Pigeon**	7	XXI (n=6) and VI (n=1)	2014-2015	Consensus of FRIAVLLLVVMTLAISAAVLV, where I27V (one strain), A28T (3 strains), A28V(one strain, V33T (one strain), M35V (one strain), A41V (one strain), V43A (one strain)	Same consensus in VII.1.1 except L74I (only in the VI strain), S75G (5 strains), G77N, and I81V.	Same consensus in II except T101S and T102I	**ICPI** 1.31 only for one strain in XXI (mesogenic in chicken). **ICPI** 1.2 and **MDT** 86h for VI strain	Rohaim et al., 2016; Sabra et al., 2017

TM: transmembrane domain (N-terminal), HR: heptad repeats

El Naggar, R.F., Rohaim, M.A., Bazid, A.H., Ahmed, K.A., Hussein, H.A., and Munir, M. (2018). Biological characterization of wild-bird-origin avian avulavirus 1 and efficacy of currently applied vaccines against potential infection in commercial poultry. Arch. Virol. 163, 2743-2755.

Mohamed, M.H., Kumar, S., Paldurai, A., Megahed, M.M., Ghanem, I.A., Lebdah, M. A., et al. (2009). Complete genome sequence of a virulent Newcastle disease virus isolated from an outbreak in chickens in Egypt. Virus Genes. 39, 234-237.

Rohaim, M.A., El Naggar, R.F., Helal, A.M., Hussein, H.A. and LeBlanc, N. (2016). Genetic characterization of pigeon paramyxovirus type 1 in Egypt. Br. j. virol, 3, 27-32.

Sabra, M., Dimitrov, K.M., Goraichuk, I.V., Wajid, A., Sharma, P., Williams-Coplin, D., et al. (2017). Phylogenetic assessment reveals continuous evolution and circulation of pigeon-derived virulent avian avulaviruses 1 in Eastern Europe, Asia, and Africa. BMC Vet. Res. 13: 291.

Table S4: Head-stalk linker region involved in fusion triggering

Species	Number of sequences included	Genotype	Year	Neutralizing epitopes at C-terminal globular head											Thermostability		Head–stalk linker region
				2-3	3			4		1/1-4	2/1-2						
				LSGCRDHSH						PDKQDYQIR		RVTRVSSSS		Mayahi and Esmaelizad, 2017	315	369	NGAENSSG
				193-201	263	287	321	332–333	356	345–353	494	513–521	569	362			115-122
**Chicken**	1	II	2005	L193S and H201P	N	D	K	EK	K	K347E	G	V514I	D	R	S	I	E118A, S120N
**Chicken**	33	VII.1.1	2011-2013		K	D	K	GK	K	R353Q only one strain	D		D	A	P	V	A117V (one strain)
**Quail**	1	VII.1.1	2016		K	D	K	GK	K		D		D	A	P	V	
**Teal**	2	VII.1.1	2016		K	D	K	GK	K		D		D	A	P	V	
**Cattle egret**	1	VII.1.1	2016		K	D	K	GK	K		D		D	G	P	V	S120N
**Pigeon**	7	XXI (n=6) and VI (n=1)	2014-2015	R197I only in the VI strain	K	D	K	GN	K	K347E and D349N/S.<br><br>K347G and D349E only in the VI strain	D	S520G in XXI strains	E only in the VI strain	G	P	V	E118A, S120N, G122R (only in VI strain)

1/1-4 linear epitopes, while 2-3, 3, 4, 2/1-2 are conformational epitopes

Residues involved in receptor recognition:

R174, I175, D198, K236, E258, Y299, Y317, E401, R416, R498, Y526, E547

1. **Residues involved in hemadsorption (HAD) ability, NA activity, and fusion promotion ability to various degrees:**
T167, G171, C172, R174, C196, D198, S202, R516, Y526, E547

2. **Residues responsible for neuraminidase (NA) activity:**
R174, I175, R416, R498

3. **Interacting HN domain with F protein (HR2): ^{124}GAPVHDPDYIGGIGKELIVDDISDVTSFY^{152}** affects the fusion activity not other HN functions.
V127I (II and VI), Y132F (II), I145T (XXI.1) or I145A (II), D147G (VI)

4. **Predicted linear B-cell epitope A266 except (II V266, VI T266).**
