# Supplementary material for: Avian Paramyxovirus Type 1 in Egypt: Epidemiology, Evolutionary Perspective, and Vaccine Approach
Source: Front Vet Sci. 2021 Jul 15;8:647462. doi: 10.3389/fvets.2021.647462 (PMC8320000; doi:10.3389/fvets.2021.647462)
Supplement: Supplementary Table 5 — Vaccination strategies in chickens (NDV). [file Data_Sheet_3.PDF]

Table S5: Vaccination strategies in chickens (NDV)

Vaccine program	Pre-challenge Abs' titer (log-2)	Challenge virus/dpv	Protection %		Virus shedding (log-10)	*reference*
			Morbidity %	Mortality %		
Live VG/GA /twice- (GII)	-	VNDV (G II)/21dpv	90%	80%	-	***Eid , 1994***
Live LaSota (G-II)	9	VNDV (G II)/14dpv	20%	80%	-	***Abdel Moneim, 2006***
Live HB1, live LaSota (GII)	-	VNDV (G II)	-	90%	-	***Mohamed, 2006***
Live HB1, live LaSota , inactivated (GII)	-		-	100%	-	
Inactivated NDV (GII)	9.1	VNDV (GVII)/28dpv	10%	80%	T (3dpc): 1.9 (73.3%)<br>T (6dpc): 3.2 (28.6%)<br>T (10dpc): 0	***Kilany et al., 2015***
Inactivated NDV (GVII)	8.2		100%	100%	T (3dpc): 1.7, T (6dpc): 1.4	
PC DNA-F/bird+ PC DNA-HN/bird (GVII) vaccine / once	2.11**	VNDV (G VII)/ 14dpv	++	30- 40%	1 dpc T : 0 C: 0<br>2 dpc T: 2.38 C: 2.5<br>4 dpc T: 3.00 C: 3.1<br>7 dpc T: 2.75 C: 2.3<br>10 dpc T: 1.72 C: 1.6	***Mohamed et al., 2016***
		NDV (GVI)/ 14dpv	++	30- 40%	1 dpc T: 1.37 C: 0<br>2 dpc T: 2.95 C: 2.8<br>4 dpc T: 3.32 C: 3.5<br>7 dpc T: 2.93 C: 2.8<br>10 dpc T: 2.27 C: 2.2	
PC DNA-F/bird+ PC DNA-HN/bird (GVII) vaccine / twice	3.56**	VNDV (G VII)/ 14dpv	+	100%	1 dpc T: 0 C: 0<br>2 dpc T: 2.05 C: 0	

					4 dpc T: 2.35 C: 2.4<br>7 dpc T: 1.82 C: 2.1<br>10 dpc T: 0 C: 0	
		NDV (GVI)/ 14dpv	+	90%	1 dpc T: 0 C: 0<br>2 dpc: T: 2.38 C:1.25<br>4 dpc: T: 2.62 C: 2.5<br>7 dpc T: 2.08 C: 2.1<br>10 dpc T: 0 C: 0	
PC DNA-F/bird+ PC DNA-HN/bird (GVII) vaccine / thrice	3.64**	VNDV (G VII)/ 14dpv	+	100%	1 dpc T: 0 C: 0<br>2 dpc T: 1.88 C: 0<br>4 dpc T: 2.22 C: 2.4<br>7 dpc T: 1.62 C: 2.0<br>10 dpc T: 0 C: 0	
		NDV (GVI)/ 14dpv	+	90%	1 dpc T: 0 C: 0<br>2 dpc T: 2.23 C: 0<br>4 dpc T: 2.65 C: 2.2<br>7 dpc T: 1.82 C: 2.1<br>10 dpc T: 0 C: 0	
Live HB1, live LaSota (G II)	5	VVNDV (SR/1976)/ 21dpv	75%	75%	-	***lebdah et al ., 2016***
Live HB1, inactivated, live LaSota (GII)	66.67		100%	100%	-	
Live LaSota- Clone30 (GII), inactivated (G-II)	7.6	VND (G VII)/ 21dpv	100%	100%	T 2dpc: 1.99 7dpc: 1.22	***Saad et al ., 2017***
		VND (G VII)/ 30dpv			T 2dpc: 0.66 7dpc: 0.63	
Live HB1, live LaSota vaccine (GII)	77.2	VNDV (G VII)/21dpv	-	76%	-	***Ahmed et al., 2017***
Live HB1vaccine + Clone 30 (GII)	58.6		-	72%	-	
Live HB1, live ND 6/10 (GII)	62.7		-	82%	-	
Monovalent inactivated LaSota vaccine (GII)	6.33	VNDV (SR/1976)/ 21dpv	100%	100%	-	***Ali et al., 2017***

Trivalent inactivated vaccine (H9N2, H5N1, and NDV (GII))	8.33		100%	100%	-	
Inactivated mucosal Gel02 AIV (H5N1)/NDV vaccine (G VIId)/ once	≤ 3	VNDV (G VII) / 21dpv	-	0%	3dpc: 4.5 7dpc: 5.1<br>10dpc: 5.2 14dpc: dead	***Ismail et al., 2018***
Inactivated mucosal Gel02 AIV (H5N1)/NDV vaccine (G VIId)/ twice	4.6		-	60%	3dpc: 4.4 7dpc: 4.7<br>10dpc: 3.9 14dpc: 3.7	
Inactivated mucosal Gel02 AIV (H5N1)/NDV vaccine (G VIId)/ once, inactivated ISA71 oil-based AIV/NDV (G VIId) vaccine/ once	8.7		-	100%	Zero (no virus shedding)	
Inactivated ISA71 oil-based AIV (H5N1)/NDV vaccine (G VIId)/ once	5.7		-	80%	3dpc: 2.5 7dpc: 2.3<br>10dpc: not detectable<br>14dpc: not detectable	
Live HB1(GII) + inactivated ND (imported GVII)/ once, LaSota/ twice (GII)	5.25	VNDV / 30 days of age	80%	100%	33.3%	***Bastami et al., 2018***
Live HB1(GII) + inactivated ND (imported GVII), Clone (GII)+ inactivated ND (imported GVII), live LaSota (GII)	6.25		+	100%	22.2%	
Live HB1/ once (GII), live LaSota/ twice (GII)	3.7		+	100%	44.4%	
Live HB1 (GII)+ inactivated ND (imported GVII)/ once/ 5day, live LaSota/ once/19 day (GII)	4.38		+	100%	22.2%	
Live HB1 (GII)+ ND (local prepared; GVII)/once, live LaSota/ twice (GII)	6.5		+	100%	22.2%	
Live HB1 (GII)+ ND (local prepared; GVII) / once, live LaSota/ once (GII)	6.2		+	100%	33.3%	
Live HB1 (GII)+ inactivated ND	4.3		+	90%	44.4%	

(imported GVII)/ once/1 day, live LaSota/ once/ 10 day (GII)						
Inactivated ND (imported GVII), live HB1 (GII), live LaSota (GII)	5.25		+	100%	44.4%	
Live HB1 (GII), inactivated ND (imported GVII), live LaSota (GII)	5		+	90%	44.4%	
Live HB1 (GII), live LaSota (GII)+ ND (local prepared; GVII) , live LaSota (GII)	4.75		+	100%	44.4%	
Live HB1/ once, live LaSota/ once (GII)	4.63		+	80%	66.6%	
Saponin-adjuvanted inactivated vaccine (G VIId)	9	NDV (G VIId) /15dpv	100%	100%	no virus shedding	***El -Dabae et al., 2018***
Live LaSota vaccine (G II) + inactivated vaccine (G VII)	8	NDV/Cattle egret/Egypt (GVII)/15dpv	+	40%	+ (virus shedding) at 4 dpc	***El Naggar et al., 2018***
		NDV/Teal/Egypt (GVII)/ 15dpv	+	60%	+ (virus shedding) at 6 dpc	
Inactivated LaSota with Chitosan Adjuvant (GII)	(II) 7<br>(VII) 6.7	GVII/28dpv	87.33%	87.33%	7dpc: 2	***Nemr, 2018***
Inactivated LaSota with Montanide Adjuvant (GII)	(II) 5.6<br>(VII) 7.5		93.66%	93.66%	No virus shedding	
Inactivated G VII with Chitosan Adjuvant (G VII)	(II) 8.6<br>(VII) 8.7		100%	100%	10dpc: 2.2	
Inactivated G VII with Montanide Adjuvant GVII)	(II) 8<br>(VII) 8		100%	100%	10dpc: 1.6	
r ND (GII), live HB1, live LaSota (G II)	6.5	NDV (GVIId)/ 28 days of age	+	53.4%	C (3dpc): 3 ***<br>C (5dpc): 1	***Ellakany et al.,***

					C (7dpc): 1	***2019b***
Live HB1, live PHY.LMV.42 (G II)	5.3		+	60%	C (3dpc): 6 ***<br>C (5dpc): 2<br>C (7dpc): 1	
Live HB1, live VG/GA (G II)	6		+	60	C (3dpc): 3 ***<br>C (5dpc): 0<br>C (7dpc): 9	
Live HB1, Live LaSota (G II)	4.6		+	93.4%	C (3dpc): 5***<br>C (5dpc): 4<br>C (7dpc): 10	
Live HB1 (G II), inactivated ND (GII)	4		+	100%	C (3dpc): 3 ***<br>C (5dpc): 0 C (7dpc): 0	
Live HB1 (GII), live ND 60-P3 strain (GII), live LaSota (GII)	4.33	VNDV (GVII)/12dpv	-	93.4%	T (3, dpc): 3.29 (100%)<br>T (5 dpc): 2.55 (100%)<br>T (7dpc): 1.68 (28.50%)<br>T (9dpc): 1.05 (1.40%)	***Ayoub et al., 2019***
Live HB1, live LaSota (GII)	4.26		-	86.7%	T (3 dpc): 3.91 (100%)<br>T (5 dpc): 2.92 (100%)<br>T (7dpc): 1.86 (77%)<br>T (9dpc): 1.25 (30.77%)	
Live HB1 (GII), live ND 60-P3 strain (GII)+ Inactivated (GII), LaSota (GII)	5.3		-	100%	T (3 dpc): 2.92 (66.60%)<br>T (5 dpc): 1.71 (66.60%)<br>T (7dpc): 1.67 (33.30%)<br>T (9dpc): 0%	
Live HB1 (GII), live ND 60-P3 strain (GII)+ Inactivated (GVII), LaSota (GII)	5.2		-	100%	T (3 dpc): 2.75 (66.60%)<br>T (5 dpc): 1.65 (66.60%)<br>T (7dpc): 1.05 (33.30%)	

					T (9dpc): 0%	
Live HB1 (GII)+ Inactivated (GVII), live ND 60-P3 strain (GII), Inactivated (GVII), live LaSota (GII)	6.13		-	100%	T (3 dpc):1.67 (66.60%)<br>T (5 dpc): 0%<br>T (7dpc): 0%<br>T (9dpc): 0%	
Live HB (GII)+ Inactivated (GII), live ND 60-P3 strain (GII), Inactivated (GII), live LaSota (GII)	6.13		-	100%	T (3 dpc): 1.86 (66.60%)<br>T (5 dpc): 0%<br>T (7dpc): 0% T (9dpc): 0%	
Live NDV vaccine (G VIId)	4.23	V NDV(G VIId)/ 9dpv	+ (weak)	75%	T (3dpc): 5.2 C (7dpc): 3.7	***Amer et al., 2019***
Live LaSota vaccine (G II)	4.82		+ (weak)	40%	T (3dpc): 5.7 C (7dpc): 5.4	
Inactivated NDV vaccine (G II)	2.5	NDV (G VII)/ 21dpv	+++/++<br>(N.S= 60%)	33.3%	2 dpc T: 8.5 C: 4<br>6 dpc T: 8.5 C: 8<br>9 dpc T: 0 C: 4	***Sedeik et al., 2019***
Inactivated NDV vaccine (GVII)	4		++/+<br>(no N.S)	53.3%	2 dpc T: 3.5 C: 0<br>6 dpc T: 8.2 C: 0<br>9 dpc T: 0 C: 8	
Bivalent Inactivated (NDV vaccine (G II)+ H5)	3.5		++/+ (no N.S)	46.7%	2 dpc T: 3 C: 4<br>6 dpc T: 5 C: 0<br>9 dpc T: 0 C: 0	
Inactivated LaSota (G II)	II: 8<br>VII: 6	NDV (G VII) / 28dpv	100%	100%	T 2 dpc: 0 4dpc : 3.66<br>T 7dpc: 6.66 10dpc: 4.33	***Mahmoud et al., 2019b***
Inactivated NDV (GVII) + inactivated LaSota (GII)	II: 10<br>VII: 9		100%	100%	T 2 dpc: 0 4dpc : 0<br>T 7dpc: 2 10dpc: 3.33	
Inactivated NDV (GVII)	II: 4<br>VII: 8		100%	100%	T 2 dpc: 5.33 4dpc: 5.33<br>T 7dpc: 6.66 10dpc:3	
rLaSota (GII)	6.2	VNDV (GII)/21dpv	100%	100%	-	***Abozeid et***

rLaSota/S- codon of variant IBV (Y1145A) (GII)	5.7–6		100%	100%	-	***al., 2019***
Live NDV V4 strain vaccine (G II), LaSota + IB (GII), Inactivated LaSota (GII)+ AI, live LaSota (GII)	6.7		+ mild	96%	3 dpc T: 30% C: 100%<br>6 dpc T: 0% C: 30%	
Live LaSota +IB (GII), Inactivated LaSota (GII)+ AI, live LaSota (GII), Live NDV V4 strain vaccine (G II)	6.9	VNDV (G VIId)/ on day 32 of age	+ mild	96%	3 dpc T: 0% C: 0%<br>6 dpc T: 0% C: 0%	***Shahin et al., 2019***
Live NDV V4 strain vaccine (G II), live LaSota+ IB (GII), Inactivated LaSota (GII)+ AI, live LaSota (GII), Live NDV V4 strain vaccine (G II)	7.1		+ mild	96%	3 dpc T: 0% C: 30%<br>6 dpc T: 0% C: 0%	
Live LaSota +IB (GII), Inactivated LaSota (GII)+ AI, live LaSota (GII)	6.2		+ mild	88%	3 dpc T: 0% C: 30%<br>6 dpc T: 30% C: 60%	
Inactivated trivalent vaccine (r H5N; clade 2.2.1.1/ clade 2.2.1.2 and LaSota-like NDV strain) (GII)	8.9	NDV (G VIId)./ 28dpv	100%	100%	3dpc: 2.1 (40%)<br>6dpc: 1.9 (13.33%)<br>10dpc: zero	***Ali et al ., 2019***
rLaSota NDV +H5- (GII)	6	VNDV (G VII)/21dpv (Single challenge)	100%	100%	3 dpc: 4.3 5 dpc: 3.2	***Said et al., 2019***
		VNDV (G VII)/21dpv (double challenge)	80%	80%	3 dpc: 5.72 5 dpc: 4.5	
Live LaSota (clone 79) (GII), WO (water in oil inactivated vaccine) (G VIIj)	5.2		100%	100%	T (3dpc): 1 C (3dpc): 0.8	
Live LaSota (clone 79) (GII), WOW (water in oil in water inactivated vaccine) (G VIIj)	5	VND (G VIIj) (VII.1.1)/ 15 dpv	100%	100%	T (3dpc): 0.8 C (3dpc): 1	***Hassan et al., 2019b***
Live LaSota (clone 79) (GII), inactivated LaSota (GII)	6.3		100%	100%	T (3dpc): 0.8 C (3dpc): 1	

Live LaSota (clone 79) (GII), LaSota (GII)	4		100%	100%	T (3dpc): 1 C (3dpc): 0.6	
Inactivated NDV vaccine (G VIId)	2.8	NDV (G VIId)/ 21dpv	Severe, score=3	0%	T (3dpc): 4.10 (100%)<br>T (5dpc): 4.00 (100%)<br>T (7dpc): 0%	***Fawzy et al., 2020***
Inactivated adjuvant NDV-ISA 70 vaccine (G VIId)	5.20		100%	100%	T (3dpc): 2.20 (40%)<br>T (5dpc): 1.80 (12 %)<br>T (7dpc): 0%	
Monovalent inactivated NDV (G VII) vaccine	9	VNDV (G VII)/ 28dpv	100%	100%	(3 dpc): 2.6<br>(5dpc): 0 (7dpc): 0	***shawky et al., 2020***
Multivalent inactivated IBV (M41+VarII) and NDV (GVII) vaccine	9.5		100%	100%	(3 dpc): 3.4 (5 dpc): 2.9<br>(7dpc): 0	
Live NDV vaccine (GII),<br>r NDV (GVII)	8.3	VNDV (G VII)/62dpv	80%	98%	T (3dpc): 3.3 (20%)<br>C (3dpc):1.6 (40%)	***Sultan et al., 2020***
Live NDV vaccine (GII),<br>Inactivated NDV (GII)	8.6		74%	98%	T (3dpc): 4.5 (60%)<br>C (3dpc): 2.3 (40%)	
Live NDV vaccine (G VIId), inactivated NDV vaccine (G VIId)	5	VNDV (G VIId)/9dpv	+<br>(mild)	80%	T (3dpc): 4.2 C (7dpc): 2	***Amer et al., 2020***
Live NDV vaccine (G II), inactivated NDV vaccine (G II)	5.2		+<br>(mild)	85%	T (3dpc): 4.7 C (7dpc): 3.6	

** Antibody titers calculated by ELIZA (log-10)

*** virus shedding was detected using inoculated embryos and hemagglutination test (log-2)

VNDV: virulent Newcastle disease virus

dpv: days post vaccination

G: genotype

dpc: days post challenge

T: tracheal shedding

C: cloacal shedding

N.S: nervous signs

References:

Abdel-Moneim, A.S., El-Sawah, A.A. and Kandil, M.A. (2006). Characterization of variant strain of Newcastle disease virus in Egypt. J. Vet. Med. Res. 16, 12-17.

Abozeid, H.H., Paldurai, A., Varghese, B.P., Khattar, S.K., Afifi, M.A., Zouelfakkar, S., et al. (2019). Development of a recombinant Newcastle disease virus-vectored vaccine for infectious bronchitis virus variant strains circulating in Egypt. Vet. Res. 50, 1-13.

Ahmed, H., Khodier, M., Kasem, S., and El-Gohary, A.E.G. (2017). Protective Efficacy of Commercial New Castle Disease Vaccines against Virulent Genotype Vii Newcastle Disease Virus. KVMJ. 15, 89-103.

Ali, A., Safwat, M., Kilany, W.H., Nagy, A., Shehata, A.A., El-Abideen, M.A.Z., et al. (2019). Combined H5ND inactivated vaccine protects chickens against challenge by different clades of highly pathogenic avian influenza viruses subtype H5 and virulent Newcastle disease virus. Vet. World. 12, 97.

Ali, Z.M., Hassan, M.A.E.M., Hussein, H.A., Ahmed, B.M., and El Sanousi, A.A.E.G. (2017). Protective efficacy of combined trivalent inactivated ISA 71 oil adjuvant vaccine against avian influenza virus subtypes (H9N2 and H5N1) and Newcastle disease virus. Vet. World. 10, 1212.

Amer, S.A.M., Ali, M.A., Kandeil, A.M., and Kutkat, M.A.A. (2019). Advancement in Vaccination of Broiler Chickens with Genotype-Matched Vaccines to Currently Epidemic Newcastle Disease Virus Genotype VII in Egypt. J. World Poult. Res. 9, 117-123

Amer, S.A.M., Maatouq, A.M., Ahmed, H.M., and Hassan, E.R. (2020). Evaluation for Efficacy of Commercially Available Vaccines Against Challenge with Newcastle Disease Virus Genotype VII in Broilers. Egypt J. Vet. Sci. 51, 35-41.

Ayoub, M.A., Elfeil, W.K., El Boraey, D., Hammam, H., and Nossair, M. A. (2019). Evaluation of some vaccination programs in protection of experimentally challenged broiler chicken against newcastle disease virus. Am. J. Anim. Vet. Sci. 14, 197-206.

Bastami, M.A., Afifi, M.A., El-Beheiry, M.A., ZouElfakar, S.A., Rafik, H.S., Ahmed, K.A., et al. (2018). Evaluation of Some Vaccination Programs Against Field Strain of Genotype VIID of Newcastle Disease in Broilers. Biosci. Res. 15, 2171-2184.

Eid, A.A. (1994). Studies on some avian viruses with special reference to a heat resistant Newcastle disease virus. Department of Poultry and Fish, Faculty of Veterinary Medicine, Zagazig University, PhD thesis, Zagazig, Egypt.

El Naggar, R.F., Rohaim, M.A., Bazid, A.H., Ahmed, K.A., Hussein, H.A., and Munir, M. (2018). Biological characterization of wild-bird-origin avian avulavirus 1 and efficacy of currently applied vaccines against potential infection in commercial poultry. Arch. Virol. 163, 2743-2755.

El-Dabae, W.H., Hussein, H.A., Rohaim, M.A., El-Safty, M.M., Ata, N.S., and Reda, I.M. (2018). Saponin-adjuvanted vaccine protects chickens against velogenic Newcastle disease virus. Arch. Virol. 163, 2423-2432.

Ellakany, H., El-Hamid, A., Nasef, S., Abdel Aziz, M., Gado, A., and Zedan, R. (2019). Evaluation of the protection of commercial live and inactivated NDV vaccines against Newcastle virus genotype VIId circulating in the field. DJVS. 1, 17-20.

Fawzy, M., Ali, R. R., Elfeil, W.K., Saleh, A.A., and El-Tarabilli, M.M.A. (2020). Efficacy of inactivated velogenic Newcastle disease virus genotype VII vaccine in broiler chickens. In Vet Res Forum, 11(2), 113. Faculty of Veterinary Medicine, Urmia University, Urmia, Iran.

Hassan, M.I., Abd El-Azeem, M.W., Selim, A., and Sultan, S. (2019). Molecular and biological characterization of the immunological potency of Newcastle disease virus oil emulsion–inactivated vaccines prepared from field isolate obtained from vaccinated chickens outbreak. Braz. J. Microbiol. 1-12.

Ismail, N.M., El-Deeb, A.H., Emara, M.M., Tawfik, H.I., Wanis, N.A., and Hussein, H.A. (2018). Prime-boost vaccination strategy against avian influenza and Newcastle disease viruses reduces shedding of the challenge viruses. VirusDisease. 29, 324-332.

Kilany, W.H., Ali, A., Bazid, A.H.I., ZainEl-Abideen, M.A., and Elsayed, M. (2015). Evaluation of two inactivated Newcastle disease virus vaccines (genotype II and VII) against challenge of Newcastle disease genotype VII infection in chicken. J. Anim. Vet. Adv. 14, 211-218.

Lebdah, M.A., Megahed, M.M., Hassanin, O.A., and Ali, A.M. (2016). The negative impact of chicken infectious anemia virus infection on immune responses to different Newcastle disease virus vaccination programs. Zag. Vet. J. 44, 138-148.

Mahmoud, N.K., El-Deeb, A.H., Emara, M.M., Abd El-Khaleck, M.A., and Hussein, H.A. (2019). Genotypes II and VIId-based inactivated Newcastle disease vaccine reduces virus shedding. VirusDisease. 30, 453-461.

Mohamed, M.H., Abdelaziz, A.M., Kumar, S., Al-Habib, M.A., and Megahed, M.M. (2016). Effect of phylogenetic diversity of velogenic Newcastle disease virus challenge on virus shedding post homologous and heterologous DNA vaccination in chickens. Avian Pathol. 45, 228-234.

Mohamed, M.H.A (2006). Current status of Newcastle disease at Sharkia governorate with special reference to characterization and diagnosis using PCR. Department of Avian and Rabbit Medicine, Faculty of Veterinary Medicine, Zagazig University, Master thesis, Zagazig, Egypt.

Nemr, M.H.M. (2018). Anti-Viral activity of nanoparticles based chemotherapeutic system. Department of Virology, Faculty of Veterinary Medicine, Suez Canal University, PhD thesis, Ismalia, Egypt.

Saad, A.M., Samy, A., Soliman, M.A., Arafa, A., Zanaty, A., Hassan, M.K., et al. (2017). Genotypic and pathogenic characterization of genotype VII Newcastle disease viruses isolated from commercial farms in Egypt and evaluation of heterologous antibody responses. Arch. Virol. 162, 1985–1994.

Said, M, Soliman, A.M., Mousa, S., Arafa, A., Hussein, A.H., Amarin, N., and Mundt, E. (2019). Efficacy of Bivalent Inactivated Vaccine Containing Insect Cell–Expressed Avian Influenza H5 and Egg-Based Newcastle Disease Virus (NDV) Against Dual Infection with Highly Pathogenic H5N1 and Velogenic NDV in Chickens. Avian Dis. 63, 474-480.

Sedeik, M.E., Elbestawy, A.R., El-Shall, N.A., Abd El-Hack, M.E., Saadeldin, I.M., and Swelum, A.A. (2019). Comparative efficacy of commercial inactivated Newcastle disease virus vaccines against Newcastle disease virus genotype VII in broiler chickens. Poult. Sci. 98, 2000-2007.

Shahin, R., Yousef, Y., and Ibrahim, M. (2019). Protective Efficacy of Some Newcastle Disease Virus (NDV) Vaccination Programs against Velogenic NDV Genotype VII in Broiler Chickens. JCVR. 1, 103-114.

Shawky, A., Ahmed, B.M., El-Makaky, H.M., and El-Sanousi, A.A. (2020). A matching multivalent vaccine candidate combining velogenic NDV genotype VII and variant IBV protects chicken from virulent challenge and eliminates virus shedding. Adv. Anim. Vet. Sci. 8, 1380-1387.

Sultan, H.A., Talaat, S., Elfeil, W.K., Selim, K., Kutkat, M.A., Amer, S.A., and Choi, K.S. (2020). Protective efficacy of the Newcastle disease virus genotype VII–matched vaccine in commercial layers. Poult. Sci. 99, 1275-1286.
