# Supplementary material for: Avian Paramyxovirus Type 1 in Egypt: Epidemiology, Evolutionary Perspective, and Vaccine Approach
Source: Front Vet Sci. 2021 Jul 15;8:647462. doi: 10.3389/fvets.2021.647462 (PMC8320000; doi:10.3389/fvets.2021.647462)
Supplement: Supplementary Table 6 — Vaccination strategies in pigeons (PPMV-1). [file Data_Sheet_4.PDF]

Table S6: Vaccination strategies in pigeons (PPMV-1)

Vaccine program	Pre-challenge Abs' titer (log-2)	Challenge virus/dpv	Protection %		Virus shedding (log-10)	*Reference*
			Morbidity	Mortality		
LaSota-live (GII), inactivated PPMV-1 (GVI)	NR	V PPMV-1 (G VI)	-	100%		***Hassan, 2005***
Inactivated PPMV-1 (G VI)				90%		
Inactivated PPMV-1 (GVI)	5.0	V PPMV-1 (G VI)/ 21dpv	-	100%	-	***Amer et al., 2013***
Live HB1(GII)	4.5		-	50%	-	
Inactivated ND vaccine (GII)	4.3		-	60%	-	
Bivalent inactivated vaccine (S. Typhimurium and PPMV-1) (GVI) with Montanide ISA 206	8	V PPMV-1 (G VI)/ 21dpv	100%	100%	-	***Khedr et al., 2016***
Inactivated PPMV-1 aluminum hydroxide gel adjuvanted vaccine (GVI) S/C	9.3	V PPMV-1(G VI)/ 21dpv		100%	C (7dpc): 1.00	***Soliman et al., 2019***
Inactivated PPMV-1 oil adjuvanted vaccine (GVI) S/C	7.8		+	70%	C (7dpc): 1.78	
Inactivated PPMV-1 aluminum hydroxide gel adjuvanted vaccine (GVI)/ IM	5.6			100%	C (7dpc): 2.08	
Inactivated PPMV-1 vaccine (GVI)/ once	5.1 (14dpv)	-				***Abotaleb et al., 2019***
Inactivated PPMV-1 vaccine (GVI)/ twice	6 (14dpv)					

V PPMV-1: velogenic pigeon paramyxovirus-1 G: genotype dpv: days post vaccination C: cloacal shedding

References:

Abotaleb, M.M., Maher, A., Fathy, M., Darwish, D.M., Elsafty, M. M., Abdelbaky, M. H., et al. (2019). Quantity of HI Antibodies in Sera of SPF Chickens Inoculated with Inactivated Commercial Pigeon Paramyxovirus, Type1 Vaccine in Comparison with Vaccinated Pigeon. Alex. J. Vet. Sci. 63, 98-102.

Amer, M.I.S., El-Bagoury, G.F., and Khodeir, M.H. (2013). Evaluation of the immune response of pigeons to Newcastle disease and pigeon paramyxo virus vaccines. BVMJ. 24, 148-156.

Hassan, E.A. (2005): Studies on protection of pigeon squabs against Infection with pigeon paramyxovirus-1 using different strains of Newcastle disease vaccine in the period before the age of vaccination With PMV-1vaccine, Egypt. J. Agric. Res. 83.

Khedr, A.A., Abdel Latef, A.A., Sayed, M.L., Abdrabo, M.A., Nagy, N., Radwan, A.A., et al. (2016). Efficacy of an Experimental Combined Inactivated Salmonella Typhimurium and Paramyxovirus Vaccine in Pigeons. Zag. Vet. J. 44, 149-155.

Soliman, Y.A., El-Nagar, E., Abd-wanees, N.A., and El-Safty, M. (2019). Efficacy of Prepared Oil Inactivated Pigeon Paramyxo Vaccine. AJVS. 60, 30-40.
